# Microenvironment-responsive injectable marine polysaccharide hydrogel empowered by bimetallic MOF nanozymes for in situ pancreatic cancer immunotherapy *via* ferroptosis-mediated cGAS-STING activation

**DOI:** 10.1016/j.mtbio.2026.103735

**Published:** 2026-10-09

**Authors:** Yihao Zhan, Chuanqin Shi, Jiqing Dong, Shihua Zhao, Xintao Gao, Junyao Li, Xiangyan Chen, Yantao Li

**Affiliations:** a State Key Laboratory of Advanced Optical Polymer and Manufacturing Technology, Qingdao Key Laboratory of Biomacromolecular Drug Discovery and Development, College of Chemical Engineering, Qingdao University of Science and Technology, Qingdao, 266042, China; b Center of Translational Medicine, Zibo Central Hospital Affiliated to Binzhou Medical University, Zibo, 255020, China

**Keywords:** MOF nanozymes, Polysaccharide hydrogels, Ferroptosis, cGAS-STING, Pancreatic cancer

## Abstract

Pancreatic cancer is characterized by a dense desmoplastic stroma and a profoundly immunosuppressive tumor microenvironment (TME), which jointly impede drug penetration and immune activation. Here, we report a microenvironment-responsive injectable marine polysaccharide hydrogel (G@ZnFe-Gel) empowered by a Zn/Fe bimetallic MOF nanozyme to achieve in situ pancreatic cancer immunotherapy *via* ferroptosis-cGAS-STING synergy. Following intratumoral injection, the hydrogel undergoes rapid thermally and Ca^2+^-reinforced gelation, forming a stable intratumoral depot that ensures prolonged local retention within stromal-dense pancreatic tumors. Mechanistically, glucose oxidase (GOD)-mediated glucose oxidation disrupts tumor metabolism while generating H_2_O_2_, which is further converted into highly reactive oxygen species through Fe^3+^-mediated Fenton-like reactions, inducing lipid peroxidation and ferroptosis. Comparisons with the Zn-Gel control further support the additional contributions of Fe incorporation and GOD loading to the enhanced ferroptosis-associated responses. In addition, ferroptosis-associated mitochondrial stress and potential DAMP release, including mtDNA, may contribute to enhanced cGAS-STING signaling, with a possible additional contribution from Zn^2+^ signaling. In a subcutaneous pancreatic cancer model, G@ZnFe-Gel markedly suppressed tumor growth, promotes macrophage reprogramming, enhanced cytotoxic T-cell infiltration, and remodeled the immunosuppressive TME without systemic toxicity. This work establishes a mechanism-driven hydrogel-nanozyme platform that integrates local retention, regulated cell death, and innate-adaptive immune crosstalk, offering a promising strategy for immunologically cold and stroma-rich solid tumors.

## Graphical abstract

**Figure:**
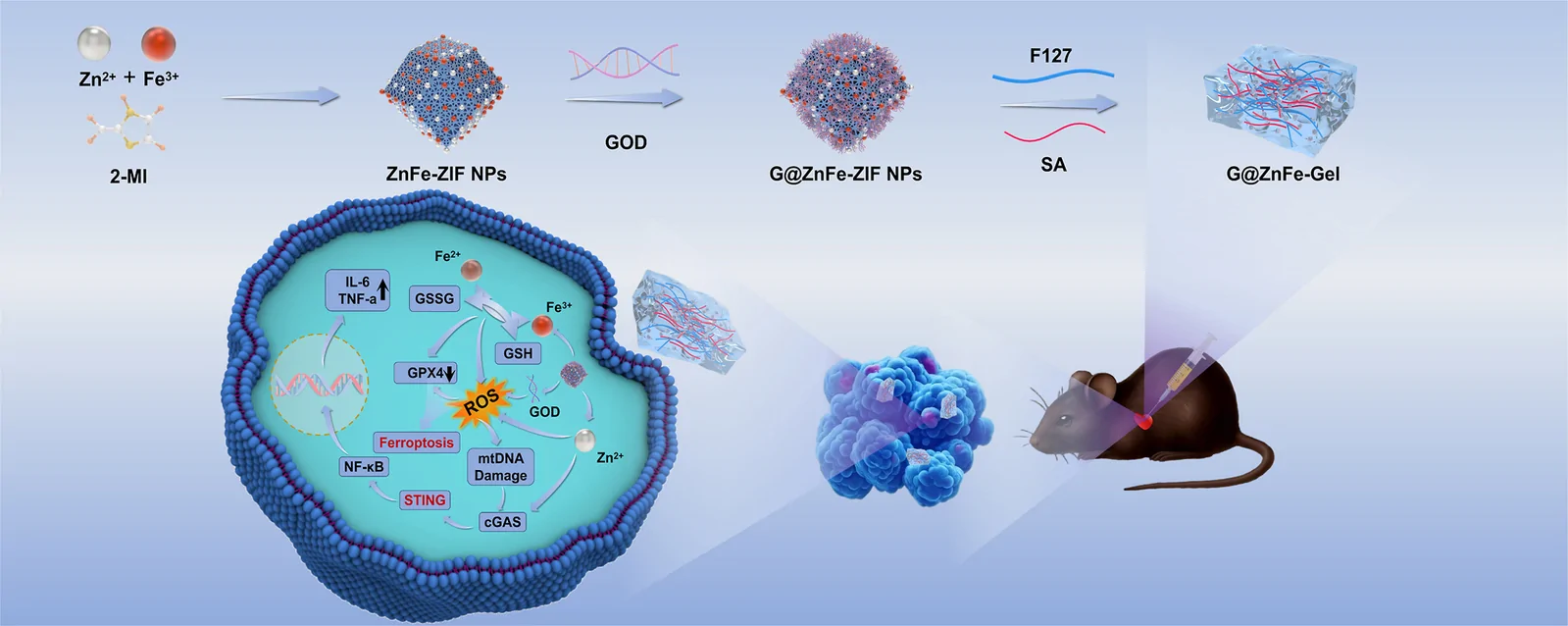

## 1 Introduction

Pancreatic cancer (PC) is a highly aggressive malignancy with a dismal prognosis, with a five-year survival rate below 10%, owing to its insidious onset, early metastasis, and pronounced resistance to chemotherapy and radiotherapy [1]. A major therapeutic barrier lies in its extensive desmoplastic tumor microenvironment (TME), dominated by activated pancreatic stellate cells and a dense extracellular matrix rich in collagen and fibronectin [2]. This stromal barrier severely impedes drug and nanomedicine penetration while restricting effector T-cell infiltration, thereby promoting therapeutic resistance and immune evasion [3]. Meanwhile, the enrichment of immunosuppressive cell populations, including tumor-associated macrophages and myeloid-derived suppressor cells, establishes an immunologically “cold” tumor phenotype, resulting in limited clinical benefits from conventional chemotherapy and immune checkpoint blockade [4]. Consequently, therapeutic strategies capable of simultaneously overcoming stromal barriers and reprogramming the immunosuppressive TME are urgently needed.

Recently, injectable hydrogels derived from marine polysaccharides including sodium alginate and chitosan, have emerged as promising platforms for localized tumor therapy due to their excellent biocompatibility, biodegradability, and structural tunability [5]. These hydrogels can be minimally invasively delivered to tumor sites and rapidly undergo in situ gelation, forming porous networks that enable local retention and sustained release of therapeutics while reducing systemic toxicity. Notably, some marine polysaccharides also exhibit intrinsic immunomodulatory properties, supporting integrated drug delivery and immune regulation [6]. Building on these merits, marine polysaccharide-based injectable hydrogels have been progressively developed into multifunctional local treatment platforms through the incorporation of exogenous bioactive components. For instance, alginate- and chitosan-derived hydrogels integrating chemotherapeutics, immune adjuvants, or catalytic nanocomponents have demonstrated the capacity to suppress postoperative tumor growth while concurrently activating antitumor immune responses in solid tumor models [7]. Despite these advances, systematic investigations of marine polysaccharide-based injectable hydrogels for in situ immunotherapy of pancreatic cancer remain limited, largely due to challenges in achieving stable local retention within dense tumor tissues and eliciting effective immune activation in an immunosuppressive microenvironment.

To enhance intratumoral accumulation and retention, increasing attention has focused on exploiting the aberrant physicochemical characteristics of the TME to enable stimulus-responsive aggregation and in situ immobilization. Tumor tissues typically exhibit mild acidity, elevated concentrations of metal ions (e.g., Ca^2+^ and Fe^2+^/Fe^3+^), reductive stress, and overexpression of proteases, which can serve as endogenous triggers for site-specific retention and activation [8]. For example, tumor microenvironment-responsive nanoplatforms can undergo enzyme- or GSH-triggered aggregation in situ, thereby enhancing intratumoral retention and therapeutic efficacy [9]. At the immunological level, effective antitumor immunity relies on coordinated innate and adaptive immune activation. The cGAS-STING pathway functions as a central innate immune sensor, recognizing cytosolic DNA to induce type I interferon production and promote antigen presentation and cytotoxic T-cell activation [10]. Ferroptosis, an iron-dependent form of regulated cell death driven by lipid peroxidation, can further amplify this process by releasing mitochondrial DNA and damage-associated molecular patterns (DAMPs) that activate the cGAS-STING axis, forming a positive feedback loop between tumor cell death and immune activation [11]. Accordingly, synergistic regulation of the ferroptosis-cGAS-STING axis represents a promising strategy to overcome immune suppression in pancreatic cancer. In this context, rational integration of exogenous functional components into marine polysaccharide hydrogels is critical for simultaneously reinforcing local retention and amplifying antitumor immune activation in situ [12].

Metal-organic frameworks (MOFs)-based nanozymes have emerged as powerful components for hydrogel-assisted cancer therapy due to their precise structural engineering and multifunctional catalytic performance [13]. When integrated into hydrogel matrices, these nanozymes enable synergistic therapeutic effects across multiple treatment levels [14]. Through supramolecular interactions such as metal coordination, hydrogen bonding, and electrostatic interactions, MOF nanozymes can reinforce the hydrogel network, thereby improving its physicochemical characteristics and mechanical robustness [15]. Meanwhile, their enzyme-mimicking catalytic activities enable the amplification of reactive oxygen species (ROS), metabolic disruption, regulated cell death, and immune activation within the TME [16]. In particular, MOF nanozymes incorporating Fe-, Cu-, or Mn- ions have been shown to induce ferroptosis or other metal-dependent cell death pathways while simultaneously promoting immune stimulation [17]. Consequently, integrating MOF nanozymes with marine polysaccharide-based injectable hydrogels establishes a rational materials design framework to exploit TME responsiveness and achieve effective in situ ferroptosis-cGAS-STING-driven immunotherapy of pancreatic cancer.

In this study, we developed a microenvironment-responsive injectable marine polysaccharide-based hydrogel incorporating a Zn/Fe bimetallic MOF nanozyme, thereby establishing a synergistic in situ immunotherapeutic strategy for pancreatic cancer based on dual regulation of the ferroptosis-cGAS-STING axis (Scheme 1). Glucose oxidase (GOD), Zn^2+^, and Fe^3+^ were co-assembled within a MOF nanostructure to generate a bimetallic nanozyme (G@ZnFe NPs) capable of metabolic modulation and immune activation. These nanozymes were subsequently embedded into a hydrogel matrix composed of sodium alginate and thermos-responsive Pluronic F127, yielding an injectable formulation (G@ZnFe-Gel). Upon intratumoral injection, the system rapidly underwent in situ gelation under physiological temperature and Ca^2+^-rich tumor microenvironment conditions, enabling stable local retention and controlled release of active components. Functionally, GOD catalyzed glucose oxidation to generate H_2_O_2_ and disrupt tumor energy metabolism, while Fe^3+^ triggered Fenton-like reactions to induce ferroptosis. The release of DAMPs during ferroptosis, possibly including mtDNA, along with Zn^2+^ signaling, may help promote cGAS-STING-associated signaling and subsequent antitumor immune responses [18]. *In vivo* studies demonstrated that G@ZnFe-Gel effectively suppressed pancreatic tumor growth, remodeled the immunosuppressive TME, and elicited robust antitumor immunity without inducing noticeable systemic toxicity. Collectively, this work presents a microenvironment-responsive in situ immunotherapy that integrates ferroptosis-mediated tumor cell death with cGAS-STING activation, offering a mechanistically driven strategy to overcome both stromal barriers and immune suppression in pancreatic cancer.

**Scheme 1:**
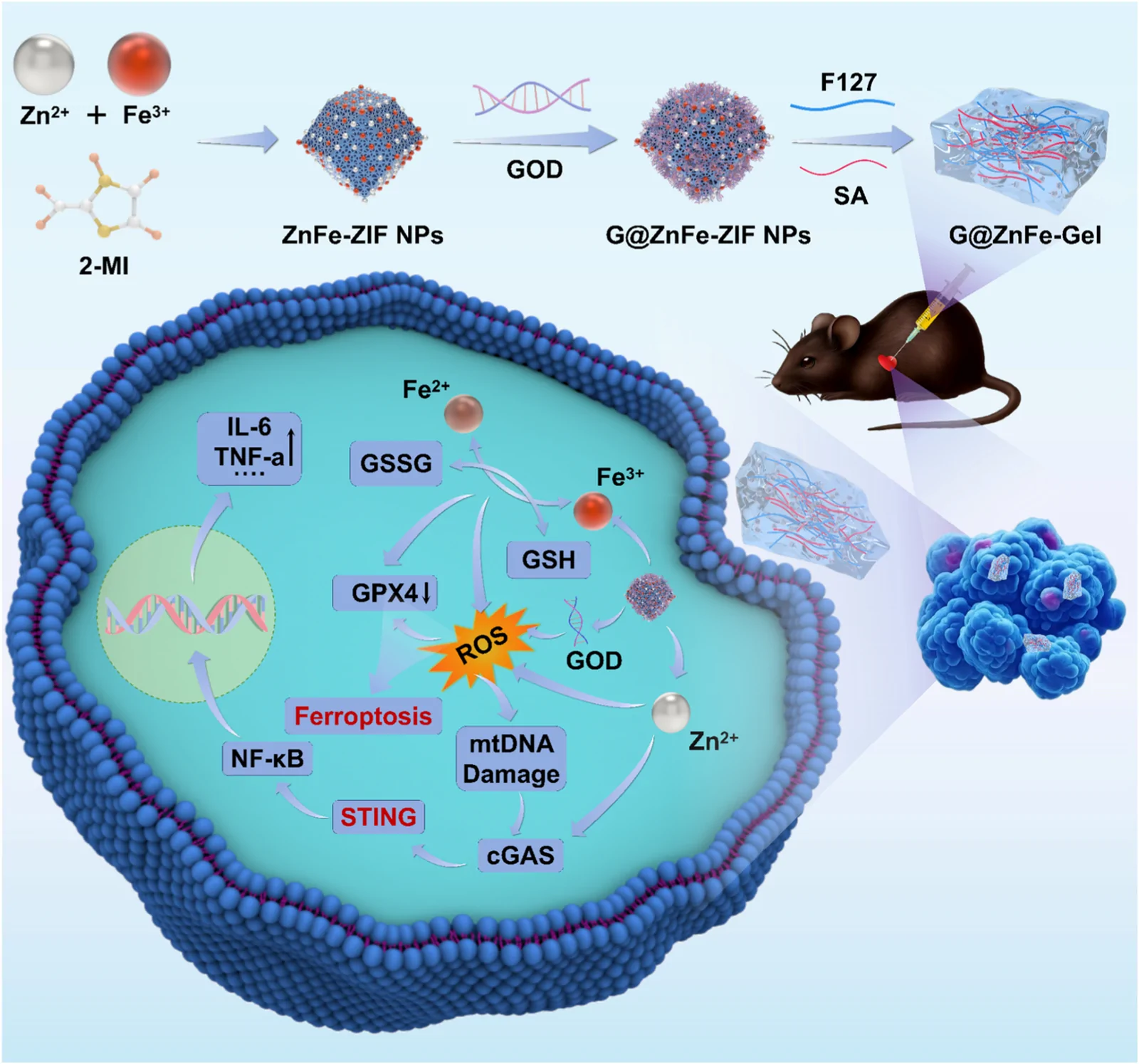
Schematic illustration of the fabrication of Bimetallic MOF Nanozymes nanozyme-powered injectable hydrogel (G@ZnFe-Gel) and its synergistic ferroptosis-cGAS-STING activation mechanism for in situ pancreatic cancer immunotherapy.

## 2 Results and discussion

### 2.1 Synthesis and characterization of G@ZnFe-ZIF NPs

As illustrated in Fig. 1a–G@ZnFe-ZIF nanozymes were successfully constructed *via* a two-step self-assembly strategy. Initially, Zn-ZIF nanoparticles (NPs) were synthesized using Zn^2+^ as metal nodes and 2-methylimidazole (2-MIM) as organic ligands. Subsequently, partial substitution of Zn^2+^ with Fe^3+^ during the coordination process yielded bimetallic ZnFe-ZIF nanoparticles with synergistic catalytic potential. Benefiting from the intrinsic porosity and abundant surface coordination sites of ZnFe-ZIF, GOD was further immobilized within the framework to afford the composite dual-metal nanozyme G@ZnFe-ZIF NPs. Transmission electron microscopy (TEM) and scanning electron microscopy (SEM) images reveal that both Zn-ZIF and ZnFe-ZIF NPs possess well-defined rhombic dodecahedral morphologies with good dispersity, showing average particle sizes of 61.9 ± 0.3 nm and 132.1 ± 0.1 nm, respectively (Fig. 1b–S1, and S2). Following GOD loading, G@ZnFe-ZIF NPs maintained their polyhedral architecture with a slightly reduced average size of 116.3 ± 0.3 nm, while the NPs surface became noticeably rougher, consistent with enzyme immobilization on the MOF surface [19]. High-angle annular dark-field scanning transmission electron microscopy (HAADF-STEM) and corresponding energy-dispersive X-ray spectroscopy (EDS) elemental mapping confirmed the homogeneous distribution of Zn and Fe throughout the NPs (Fig. 1c), verifying successful Fe^3+^ incorporation into the ZIF lattice. X-ray diffraction (XRD) patterns of ZnFe-ZIF NPs closely matched the simulated ZIF-8 diffraction peaks (Fig. 1d), demonstrating that Fe^3+^ substitution did not disrupt the original MOF topology or crystallinity. This structural preservation can be attributed to the comparable ionic radii of tetrahedrally coordinated Fe^3+^ (0.63 Å) and Zn^2+^ (0.74 Å), enabling lattice-compatible metal substitution [20]. After GOD incorporation, the XRD pattern of G@ZnFe-ZIF NPs remains essentially identical to that of ZnFe-ZIF NPs, indicating preservation of the MOF lattice integrity and crystallinity. In addition, Ultraviolet-visible (UV-Vis) absorption spectra further validated GOD incorporation (Fig. 1e). Free GOD exhibited a characteristic absorption band at 270–280 nm arising from π–π* transitions of aromatic amino acids, whereas Zn-ZIF and ZnFe-ZIF NPs showed no such feature. In contrast, G@ZnFe-ZIF NPs displayed a pronounced absorption peak around 270 nm, confirming successful enzyme loading without compromising MOF structural integrity.

**Fig. 1:**
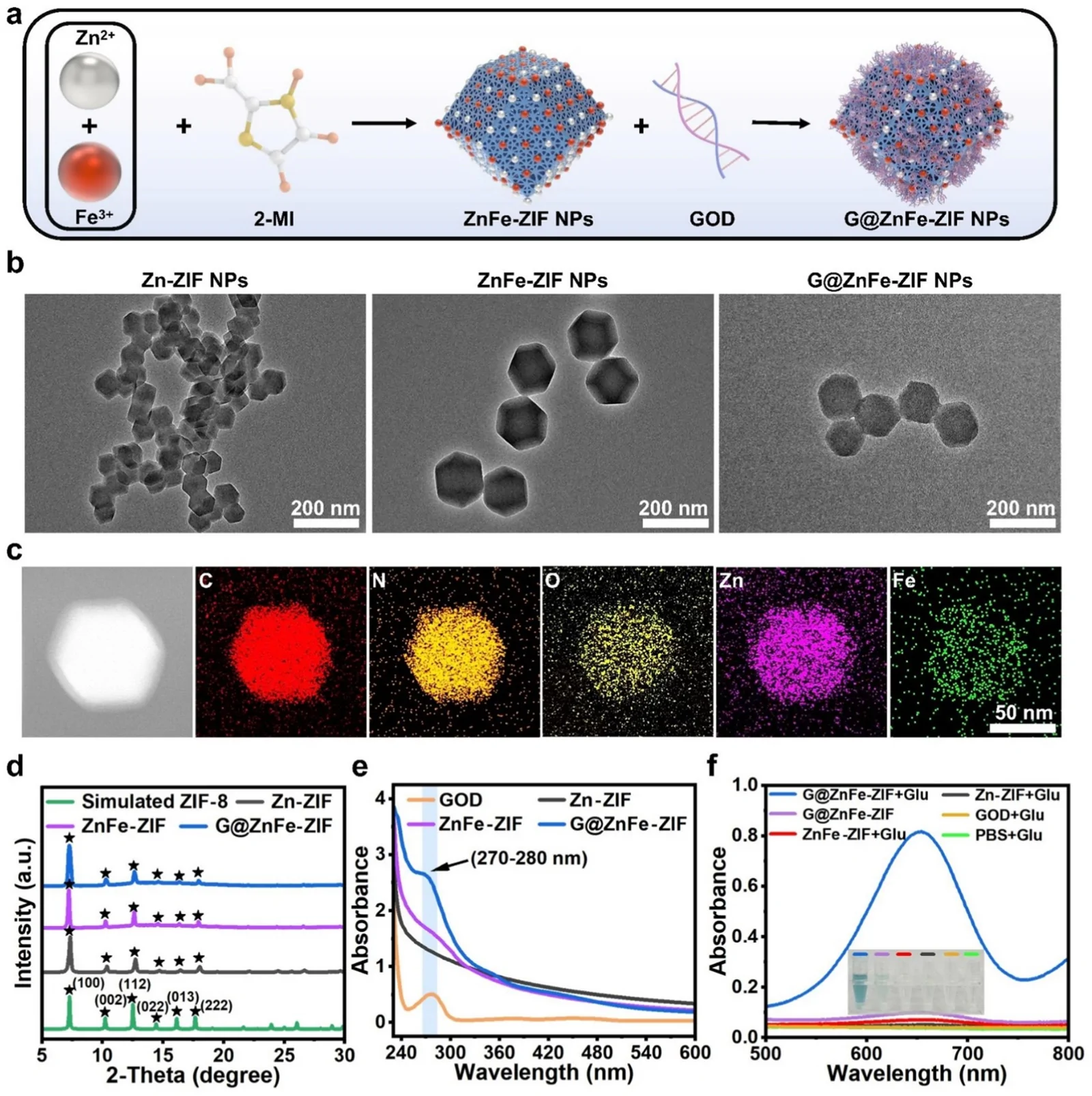
Construction and characterization of G@ZnFe-ZIF NPs. (a) Schematic illustration of the two-step self-assembly process for the fabrication of G@ZnFe-ZIF NPs. (b) TEM images of Zn-ZIF NPs, ZnFe-ZIF NPs, and G@ZnFe-ZIF NPs. (c) HAADF-STEM image of G@ZnFe-ZIF NPs and the corresponding EDS elemental mapping. (d) Simulated and experimental XRD patterns of ZIF-8, Zn-ZIF NPs, ZnFe-ZIF NPs, and G@ZnFe-ZIF NPs. (e) UV–vis absorption spectra of the different nanoparticle formulations. (f) Visible absorption spectra of the TMB-based cascade catalytic reaction together with corresponding photographs illustrating the color changes of different reaction systems. (For interpretation of the references to color in this figure legend, the reader is referred to the Web version of this article.)

The cascade catalytic activity of G@ZnFe-ZIF NPs was evaluated using 3,3′,5,5′-tetramethylbenzidine (TMB) as a chromogenic substrate. Time-dependent studies identified 30 min as the optimal incubation time, at which the characteristic absorbance of oxidized TMB reached its maximum, indicating the highest cascade efficiency (Fig. S3). Under these conditions, comparative catalytic assays showed negligible absorbance at 652 nm for PBS, ZnFe-ZIF NPs, GOD + glucose, or ZnFe-ZIF NPs + glucose groups, demonstrating their inability to independently generate hydroxyl radicals (Fig. 1f). In contrast, only the G@ZnFe-ZIF NPs + glucose group exhibited strong absorbance at 652 nm, indicative of TMB oxidation and efficient cascade catalysis. Glucose consumption assays further confirmed that GOD retained its glucose-consuming activity following immobilization and exhibited improved activity retention during prolonged incubation under serum-containing conditions (Fig. S4). Together with the structural characterization, these findings demonstrate successful enzyme immobilization, preserved catalytic function, and robust cascade activity, supporting the subsequent incorporation of G@ZnFe-ZIF NPs into injectable hydrogels for ferroptosis-driven immunotherapy.

### 2.2 Fabrication and characterization of G@ZnFe-Gel

In view of the dense stromal architecture and immunosuppressive microenvironment characteristic of pancreatic cancer, which demand prolonged local retention and sustained immune activation [21], we developed a triple-synergistic strategy integrating thermally triggered gelation, ion-mediated crosslinking reinforcement, and nanozyme-induced network densification to construct an injectable hydrogel composite (G@ZnFe-Gel).

Inverted vial tests demonstrated that hydrogels composed of SA and thermos-responsive Pluronic F127 (FS-Gel), and nanozyme-reinforced hydrogels (Zn-Gel, ZnFe-Gel, and G@ZnFe-Gel), all exhibited a free-flowing sol state at 4°C (Fig. 2a and S5). Upon increasing the temperature to above 30°C, these systems rapidly transformed into stable gel states within approximately 3 min, confirming their temperature-responsive gelation behavior. For the FS-Gel precursor, the introduction of Ca^2+^ markedly shortened the gelation time to about 2 min (Fig. S6). This result reflects a pronounced crosslinking reinforcement arising from ionic interactions between SA and Ca^2+^, which acts in concert with the thermally induced micellization of F127 to promote rapid gel formation. By contrast, G@ZnFe-Gel exhibited substantially faster gelation kinetics, with the gelation time further reduced to 1 min upon Ca^2+^ addition (Fig. 2b). This enhanced gelation performance is likely attributed to the incorporation of bimetallic MOF nanozymes, which increase network density through multivalent metal coordination and structural filling effects, synergistically accelerating hydrogel formation [22]. Moreover, as shown in Fig. 2c–G@ZnFe-Gel could be smoothly extruded through a conventional syringe and rapidly recovered its gel state under physiological temperature and local Ca^2+^-rich conditions, demonstrating excellent injectability and in situ solidification capability. These features provide distinct advantages for precise intratumoral administration and rapid local retention.

**Fig. 2:**
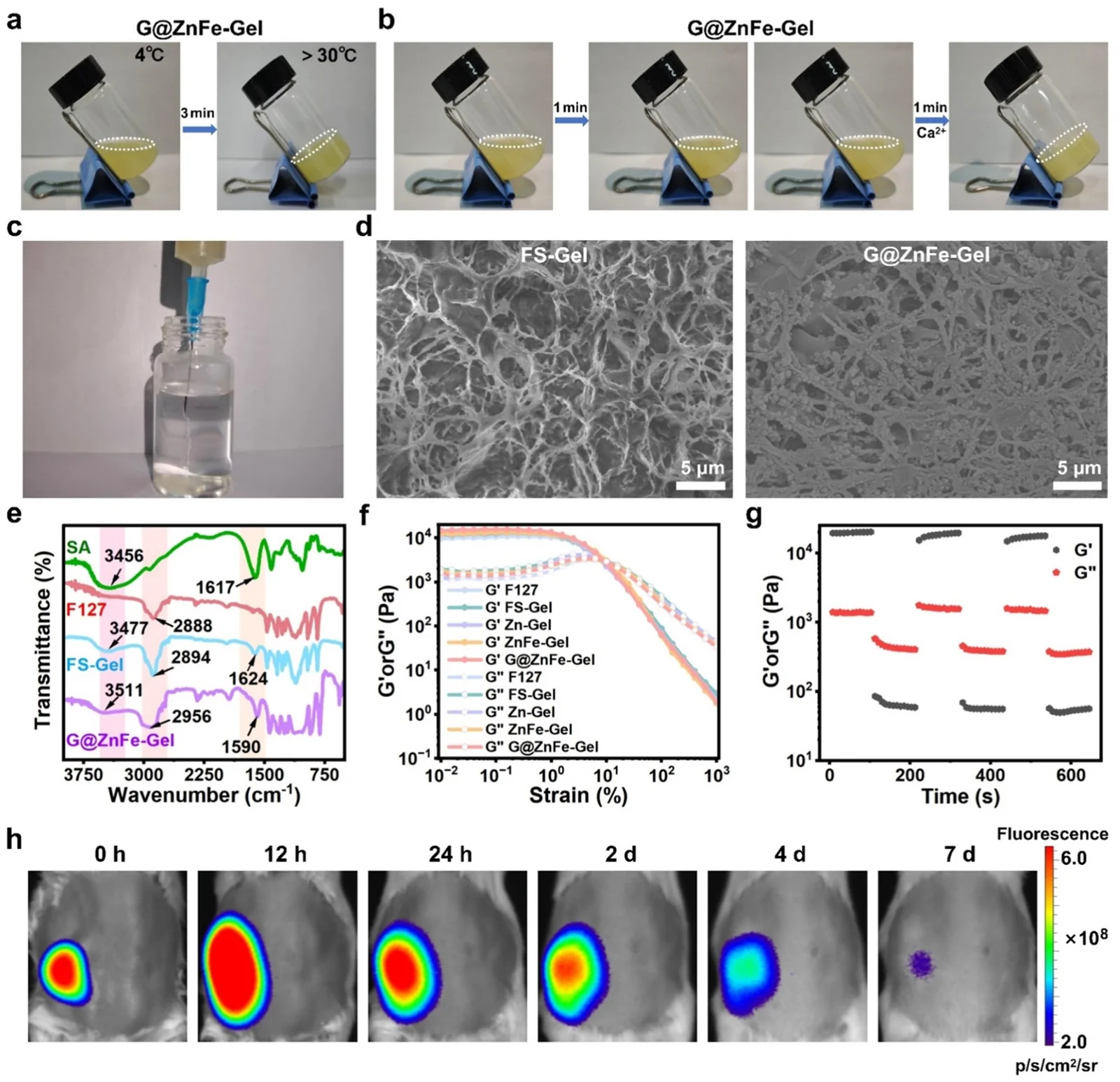
Construction and physicochemical characterization of G@ZnFe-Gel. (a) Schematic illustration of the gelation mechanism of G@ZnFe-Gel. (b) Schematic representation of the Ca^2+^-responsive gelation behavior of G@ZnFe-Gel. (c) Photograph demonstrating the injectability of G@ZnFe-Gel. (d) SEM images of FS-Gel and G@ZnFe-Gel. (e) FT-IR spectra of SA, Pluronic F127, FS-Gel, and G@ZnFe-Gel. (f) Strain sweep rheological profiles of different hydrogel formulations. (g) Time-dependent rheological behavior of G@ZnFe-Gel over 0-600 s. (h) *In vivo* retention and controlled degradation behavior of G@ZnFe-Gel in mice from 0 h to 7 days after injection.

To systematically elucidate the regulatory effect of bimetallic MOF nanozyme incorporation on the microstructure and intermolecular interactions of the hydrogel network, the microscopic morphologies of nanozyme-free FS-Gel and G@ZnFe-Gel were first comparatively analyzed. As shown in Fig. 2d, SEM images reveal that FS-Gel exhibits a relatively loose three-dimensional porous architecture with a broad pore-size distribution and limited crosslinking junctions. While upon incorporation of G@ZnFe-ZIF NPs, the hydrogel network became markedly more compact, with reduced pore sizes and a more uniform distribution. This structural evolution suggests that the bimetallic MOF nanozymes act as effective “structural bridging nodes”, enhancing network crosslinking density and overall hydrogel stability through multivalent interactions [23].

Further insight into the intermolecular interactions within the hydrogel network was obtained by Fourier-transform infrared (FT-IR) spectroscopy of individual components and composite hydrogels (Fig. 2e). SA exhibited a broad and intense absorption band at 3456 cm^−1^, attributable to O–H stretching vibrations, together with a characteristic peak at approximately 1617 cm^−1^ corresponding to the asymmetric stretching of carboxylate groups. Pluronic F127 displayed a –CH_2_ stretching vibration at 2888 cm^−1^ and a pronounced C–O–C stretching band at 1114 cm^−1^. The FT-IR spectrum of FS-Gel retained the characteristic absorption features of both SA and F127, confirming their successful integration into a stable polysaccharide-thermo-responsive polymer network. Upon incorporation of G@ZnFe-ZIF NPs, the FT-IR spectrum of G@ZnFe-Gel showed distinct peak shifts and intensity variations compared with FS-Gel, indicative of altered molecular interactions. Specifically, the O–H stretching band shifted from 3477 cm^−1^ in FS-Gel to 3511 cm^−1^ in G@ZnFe-Gel, suggesting a reconstruction of the hydrogen-bonding network induced by nanozyme incorporation. Concurrently, the –CH_2_ stretching vibration shifted from 2894 to 2956 cm^−1^, implying restricted polymer chain mobility after G@ZnFe-ZIF NPs are embedded as physical crosslinking nodes, thereby increasing network compactness [24]. In addition, the absorption band associated with carboxyl groups was displaced from 1624 to 1590 cm^−1^, which could be attributed to coordination interactions between Zn^2+^/Fe^3+^ and carboxylate groups of SA or amide groups within GOD [25]. Briefly, these concerted spectral shifts provide strong evidence that G@ZnFe-ZIF NPs are effectively integrated into the hydrogel network and jointly remodel its microstructure through metal coordination, hydrogen bonding, and physical confinement effects.

Rheological analyses provided additional insight into the mechanical properties of the hydrogel systems. As displayed in Fig. 2f, strain-sweep measurements showed that the storage modulus (G′) of G@ZnFe-Gel was consistently higher than that of FS-Gel, Zn-Gel, and ZnFe-Gel, indicating that incorporation of G@ZnFe-ZIF NPs markedly enhanced internal network stability and improved resistance to compression and deformation under external stress. Dynamic time-sweep tests revealed that G@ZnFe-Gel rapidly recovered its original G′ and G″ values upon reducing the strain from 1000% to 1% after structural disruption, and this recovery behavior remained stable over multiple cycles (Fig. 2g), demonstrating excellent self-healing capability. Such resilience is advantageous for maintaining long-term structural integrity under complex mechanical stresses *in vivo*. Frequency-sweep measurements further showed that G′ consistently exceeded G″ over the range of 0.1–20 Hz and exhibited minimal frequency dependence, confirming the predominantly elastic nature of the hydrogel network (Fig. S7). The *in vivo* degradation behavior was then evaluated using fluorescence-labeled G@ZnFe-Gel. After subcutaneous injection into Sprague-Dawley rats, the local fluorescence signal gradually decreased and nearly disappeared within 7 days (Fig. 2h and S8), revealing stable local retention followed by progressive degradation. This controlled degradation profile is favorable for minimizing long-term tissue burden while maintaining therapeutic functionality during the treatment window.

In short, the integrated thermos-ionic-nanozyme reinforcement design endows G@ZnFe-Gel with superior injectability, mechanical resilience, rapid in situ gelation, and programmable degradation, establishing a structurally robust intratumoral depot capable of sustained therapeutic release.

### 2.3 Biocompatibility assessment and ROS-driven cytotoxic mechanism of G@ZnFe-Gel in PAN02 cells

Based on these physicochemical features, the biosafety and antitumor performance of G@ZnFe-Gel were systematically evaluated. Hemocompatibility was first assessed by hemolysis assays to examine interactions between different hydrogel formulations and red blood cells (Fig. 3a). All tested groups, including PBS, FS-Gel, Zn-Gel, ZnFe-Gel, and G@ZnFe-Gel, exhibited negligible hemolysis (<5%), meeting the ISO 10993 standard for blood compatibility and indicating favorable safety profiles for potential *in vivo* administration [26]. The cytotoxic effects of different hydrogel systems against pancreatic cancer PAN02 cells were subsequently quantified using the MTT assay (Fig. 3b). Compared with FS-Gel, Zn-Gel, ZnFe-Gel, and G@ZnFe-Gel displayed concentration-dependent inhibition over the tested range of 1-16 mg mL^−1^. Notably, G@ZnFe-Gel consistently exhibited the strongest cytotoxic activity at equivalent concentrations, highlighting the superior antitumor efficacy of the nanozyme-integrated hydrogel system.

**Fig. 3:**
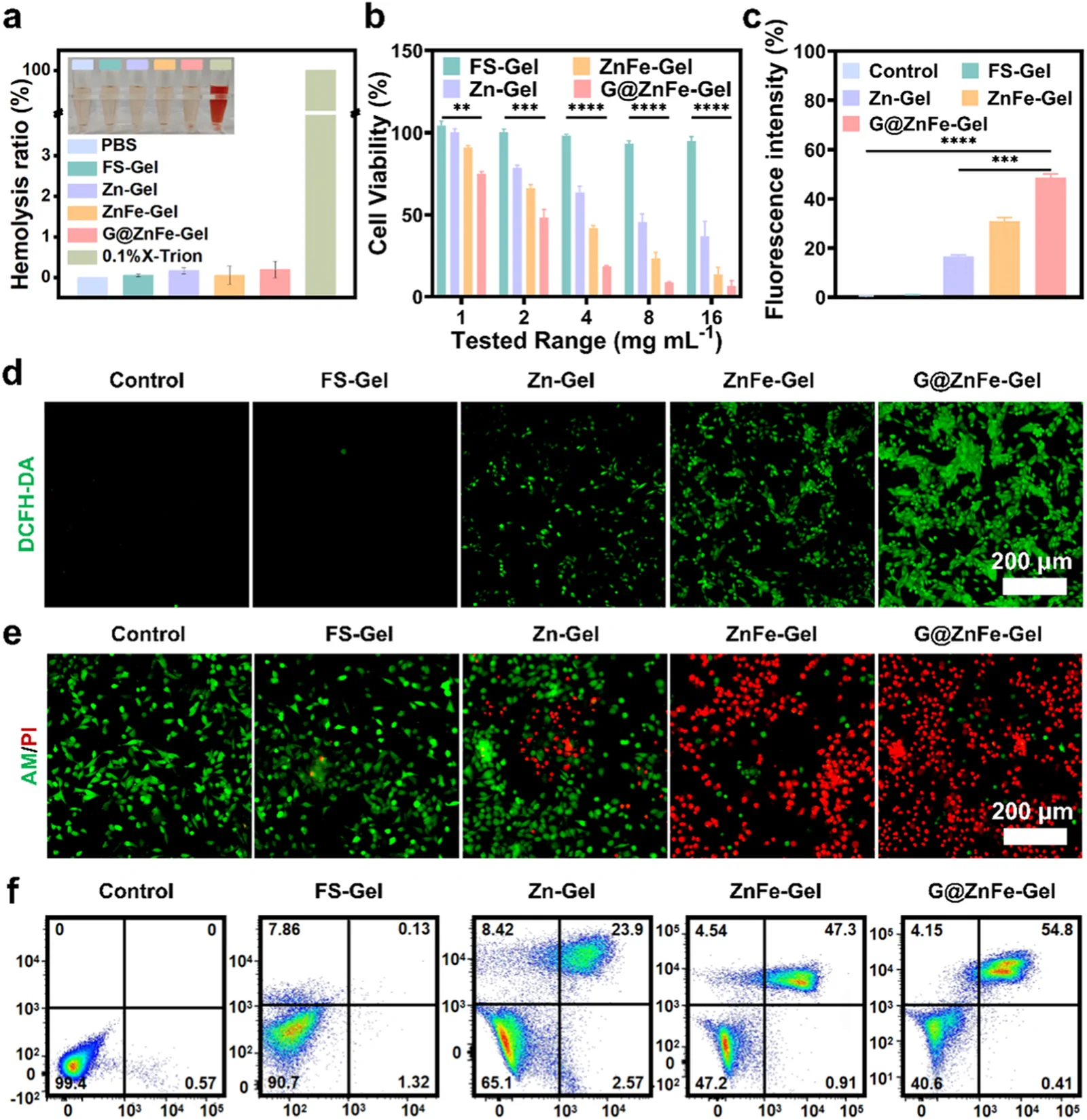
Blood compatibility of G@ZnFe-Gel and its *in vitro* induction of oxidative stress and cell death. (a) Quantitative hemolysis ratios and representative photographs of red blood cells incubated with different formulations. PBS and 0.1% Triton X-100 were used as the negative and positive controls, respectively. (b) Cell viability of PAN02 cells after treatment with different hydrogel formulations at concentrations ranging from 1 to 16 mg mL^−1^. (c) Quantitative analysis of intracellular reactive oxygen species (ROS) levels in PAN02 cells detected using the DCFH-DA probe. (d) Representative fluorescence microscopy images of intracellular ROS generation in PAN02 cells stained with DCFH-DA after different treatments. (e) Representative fluorescence microscopy images of PAN02 cells subjected to Calcein-AM/PI live/dead staining following different treatments. (f) Flow cytometric analysis of apoptosis and necrosis in PAN02 cells subjected to different treatments. ***P < 0.001, ****P < 0.0001. (For interpretation of the references to color in this figure legend, the reader is referred to the Web version of this article.)

To elucidate the underlying antitumor mechanism, intracellular ROS levels were monitored using the DCFH-DA probe (Fig. 3c, d and S9). Cells treated with Zn-Gel showed weak green fluorescence, indicating limited ROS generation, whereas ZnFe-Gel induced noticeably stronger fluorescence due to Fe-mediated ROS production. In comparison, G@ZnFe-Gel treatment resulted in the most intense fluorescence signal, suggesting a pronounced increase in intracellular ROS levels upon incorporation of GOD. Quantitative fluorescence analysis further confirmed that G@ZnFe-Gel significantly elevated intracellular ROS compared with all other groups.

The *in vitro* therapeutic efficacy was further corroborated by Calcein-AM (live, green)/PI (dead, red) dual staining assays (Fig. 3e). Among all groups, G@ZnFe-Gel-treated cells exhibited the strongest red fluorescence, indicating the highest level of cell death, in line with the enhanced ROS production. Flow cytometric analysis provided quantitative insight, revealing that G@ZnFe-Gel induced apoptosis in approximately 55.2% of PAN02 cells, with a marked increase in late-stage apoptosis (54.8%) and a concomitant reduction in viable cells to 40.6% (Fig. 3f and S10). All these findings demonstrate that G@ZnFe-Gel amplifies intracellular ROS generation through GOD-mediated glucose oxidation coupled with Fe^3+^/Zn^2+^-assisted Fenton-like cascade reactions [27], thus disrupting redox homeostasis and effectively triggering pronounced programmed cell death in pancreatic cancer cells.

### 2.4 *In vitro* evaluation of ferroptosis and cGAS-STING pathway activation

To systematically elucidate the molecular mechanisms underlying the superior antitumor efficacy of G@ZnFe-Gel, we first investigated the expression of core ferroptosis-regulatory proteins in PAN02 cells *via* Western blot. Among these proteins, glutathione peroxidase 4 (GPX4) detoxifies lipid peroxides, and its depletion compromises cellular antioxidant defenses, whereas ferritin heavy chain 1 (FTH1) regulates intracellular iron storage, with reduced FTH1 expression potentially increasing the labile iron pool [28,29]. As illustrated in Fig. 4a and S11, FS-Gel reduced FTH1 expression without decreasing GPX4 and exerted only limited effects on cell viability, ROS generation, and cell death (Fig. 3b–e), providing insufficient evidence of nonspecific ferroptosis induced by the blank hydrogel [30]. Comparison of Zn-Gel, ZnFe-Gel, and G@ZnFe-Gel revealed more pronounced reductions in GPX4 and FTH1 following Fe incorporation and GOD loading, with marked downregulation observed in the G@ZnFe-Gel group. These changes, together with the observed oxidative damage and cell death, support enhanced ferroptosis-associated responses to the combined formulation. Furthermore, we observed a significant upregulation of the cystine/glutamate antiporter xCT (SLC7A11) and the master antioxidant transcription factor NRF2, accompanied by a concomitant decrease in its negative regulator KEAP1. This coordinated NRF2-KEAP1-xCT response signifies the activation of a compensatory antioxidant program triggered by the overwhelming oxidative stress imposed by the bimetallic MOF nanozymes. Despite this adaptive response, sustained lipid peroxidation and progressive cell death persisted, indicating that the cascade catalytic pressure generated by G@ZnFe-Gel ultimately overwhelms cellular redox homeostasis and enforces ferroptosis. Given that ferroptosis-associated damage can promote the release of DAMPs, particularly mitochondrial DNA (mtDNA), we next assessed the activation status of the cGAS-STING innate immune signaling pathway [31]. As shown in Fig. 4b and S12, although the total protein levels of STING, TBK1, and IRF3 exhibited a modest decrease following treatment, their phosphorylated forms (p-STING, p-TBK1, and p-IRF3) were markedly elevated in the G@ZnFe-Gel group. This distinctive pattern, characterized by reduced total protein abundance but enhanced phosphorylation, represents a hallmark of robust cGAS-STING pathway activation [32].

**Fig. 4:**
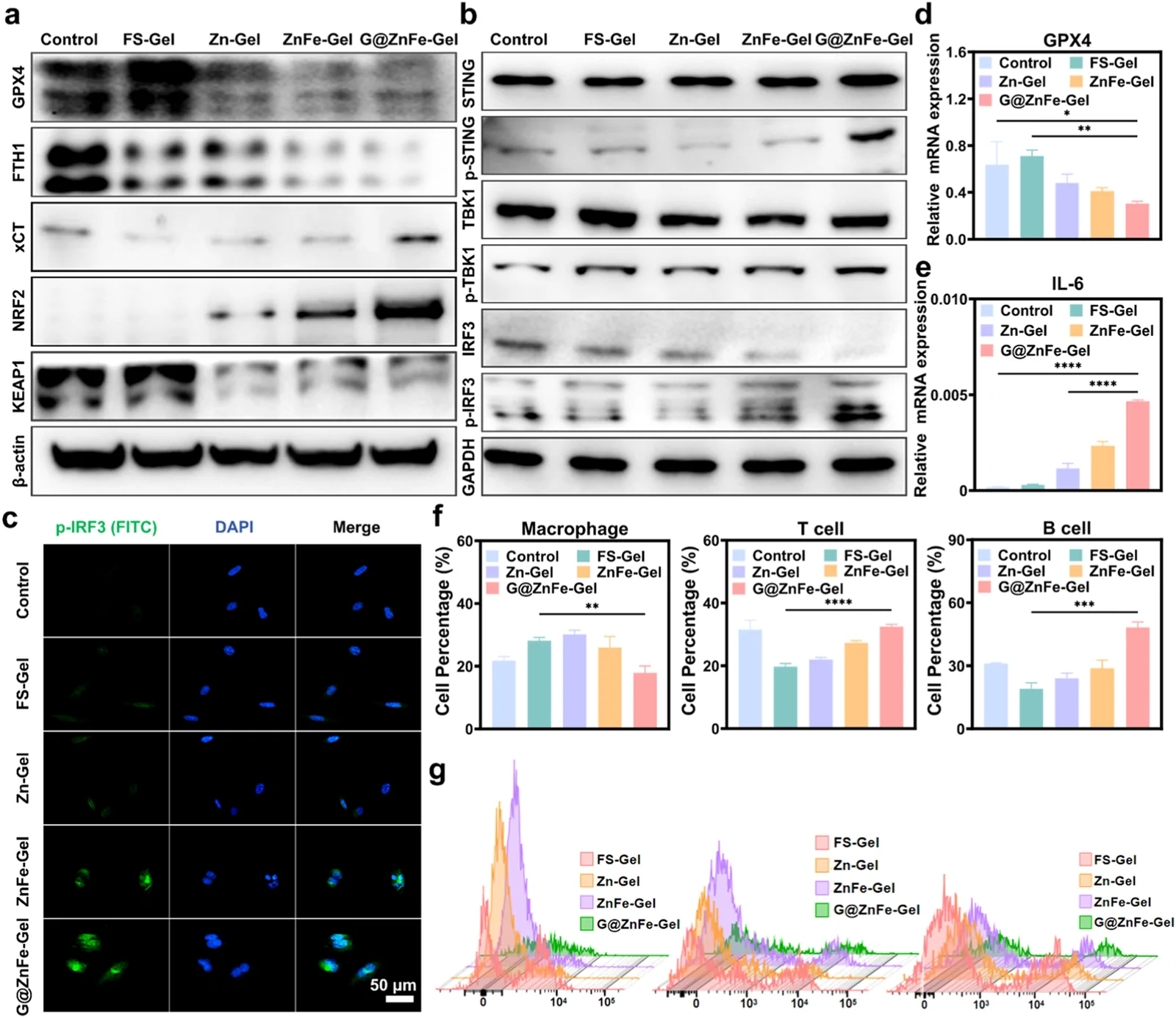
G@ZnFe-Gel synergistically induces ferroptosis and activates the cGAS-STING pathway to remodel the tumor immune microenvironment. (a) Western blot analysis of ferroptosis-related proteins (KEAP1, NRF2, xCT, FTH1, and GPX4) in PAN02 cells treated with different hydrogel formulations. (b) Western blot analysis of key proteins involved in the cGAS-STING signaling axis (p-STING, STING, p-TBK1, TBK1, p-IRF3, and IRF3) in PAN02 cells after different treatments. (c) High-magnification confocal fluorescence images showing the subcellular localization of p-IRF3 in macrophages following different treatments. Cell nuclei were stained with DAPI (blue), and p-IRF3 was stained with FITC (green). (d) Quantitative qRT-PCR analysis of GPX4 mRNA expression in PAN02 cells under different treatments (β-actin as the internal control). (e) Quantitative qRT-PCR analysis of IL-6 mRNA expression in PAN02 cells under different treatments (β-actin as the internal control). (f) Schematic illustration and representative data showing changes in peripheral immune phenotypes in tumor-bearing mice following intraperitoneal administration of different hydrogel formulations. (g) Flow cytometric quantification of macrophages, T cells, and B cells in peripheral blood. *P < 0.05,**P < 0.01,***P < 0.001,****P < 0.0001. (For interpretation of the references to color in this figure legend, the reader is referred to the Web version of this article.)

To functionally validate cGAS-STING pathway activation, we examined the nuclear translocation of phosphorylated IRF3 (p-IRF3) in macrophages (Fig. 4c). In the Control, FS-Gel, and Zn-Gel groups, p-IRF3 signals were predominantly cytoplasmic, indicating an inactive signaling state. In contrast, treatment with ZnFe-Gel, and most notably G@ZnFe-Gel, induced a clear and significant nuclear translocation of p-IRF3, indicating enhanced downstream STING-TBK1-IRF3 signaling. Given that nuclear translocation of p-IRF3 is essential for type I interferon transcription [33,34], we further quantified IFN-β expression by RT-qPCR. Consistently, G@ZnFe-Gel markedly increased IFN-β mRNA expression (Fig. S13). Together, these results support functional activation of the downstream STING-TBK1-IRF3-IFN-I signaling cascade by G@ZnFe-Gel.

The functional interplay between ferroptosis-associated stress and cGAS–STING signaling was investigated through pharmacological rescue experiments. Treatment with either the ferroptosis inhibitor Fer-1 or the cGAS inhibitor RU.521 partially restored PAN02 cell viability following G@ZnFe-Gel exposure (Fig. S14). Notably, Fer-1 attenuated treatment-induced TBK1 phosphorylation while leaving total TBK1 abundance largely unchanged (Fig. S15), accompanied by reduced IFN-β and IFIT3 transcript levels (Fig. S16). These complementary findings support a functional contribution of ferroptosis-associated stress to TBK1 activation and the downstream interferon-associated transcriptional response, with mtDNA release representing a potential molecular link to cGAS-STING signaling.

To further validate the findings at the transcriptional level, we performed real-time quantitative PCR (qRT-PCR) analysis. Results revealed a marked reduction in GPX4 mRNA levels in the G@ZnFe-Gel-treated group (Fig. 4d), consistent with Western blot results, further supporting the activation of the ferroptosis program. Additionally, the mRNA expression of the pro-inflammatory cytokine IL-6, was markedly increased in the G@ZnFe-Gel group (Fig. 4e), indicating an enhanced pro-inflammatory response accompanying the observed STING-TBK1-IRF3 signaling changes [35].

Finally, to determine whether the local immunomodulation triggered by G@ZnFe-Gel could translate to a systemic level, we analyzed the distribution of major immune cell subsets in the peripheral blood of tumor-bearing mice using flow cytometry (Fig. 4f and g). Compared with the control group, G@ZnFe-Gel treatment induced a pronounced reshaping of the systemic immune landscape. Specifically, the proportions of circulating T cells and B cells were both significantly increased in the G@ZnFe-Gel-treated mice, indicating effective activation and expansion of adaptive immune populations at the systemic level. By contrast, a modest reduction in the proportion of circulating macrophages was observed in the G@ZnFe-Gel-treated group, which may be associated with enhanced recruitment and infiltration of these cells into the tumor microenvironment. These results demonstrated that G@ZnFe-Gel-mediated in situ therapy not only remodeled the local tumor immune microenvironment, but also initiated a coordinated systemic immune response, characterized by amplified adaptive immunity and dynamic redistribution of innate immune cells.

In summary, G@ZnFe-Gel induced ferroptosis in pancreatic cancer through metabolic disruption-driven ROS amplification and Fe^3+^-mediated lipid peroxidation, while concurrently activating the cGAS-STING pathway *via* ferroptosis-associated DAMPs release and Zn^2+^ signaling. This ferroptosis-cGAS-STING synergy couples direct tumor cytotoxicity with robust innate-to-adaptive immune activation, effectively overcoming the stromal and immunosuppressive barriers characteristic of pancreatic cancer.

### 2.5 Transcriptomic analysis reveals the molecular mechanisms underlying G@ZnFe-Gel induced cell death

To systematically elucidate how the rational design of G@ZnFe-Gel is translated into intracellular biological responses in pancreatic cancer cells, RNA sequencing (RNA-seq) was performed on PAN02 cells following treatment. Consistent with the proposed “metabolic disruption-ferroptosis-cGAS-STING” synergistic regulatory framework, G@ZnFe-Gel induced a highly characteristic transcriptional reprogramming profile that tightly coupled redox imbalance, regulated cell death, and innate immune activation. Differential expression analysis revealed extensive gene upregulation and downregulation in the G@ZnFe-Gel-treated group compared with the PBS group (Fig. 5a), indicating treatment-associated transcriptional changes. The heatmap further illustrated the expression patterns of selected differentially expressed genes across the two groups (Fig. 5b), consistent with the transcriptional effects of the G@ZnFe-ZIF NPs embedded within the marine polysaccharide hydrogel matrix.

**Fig. 5:**
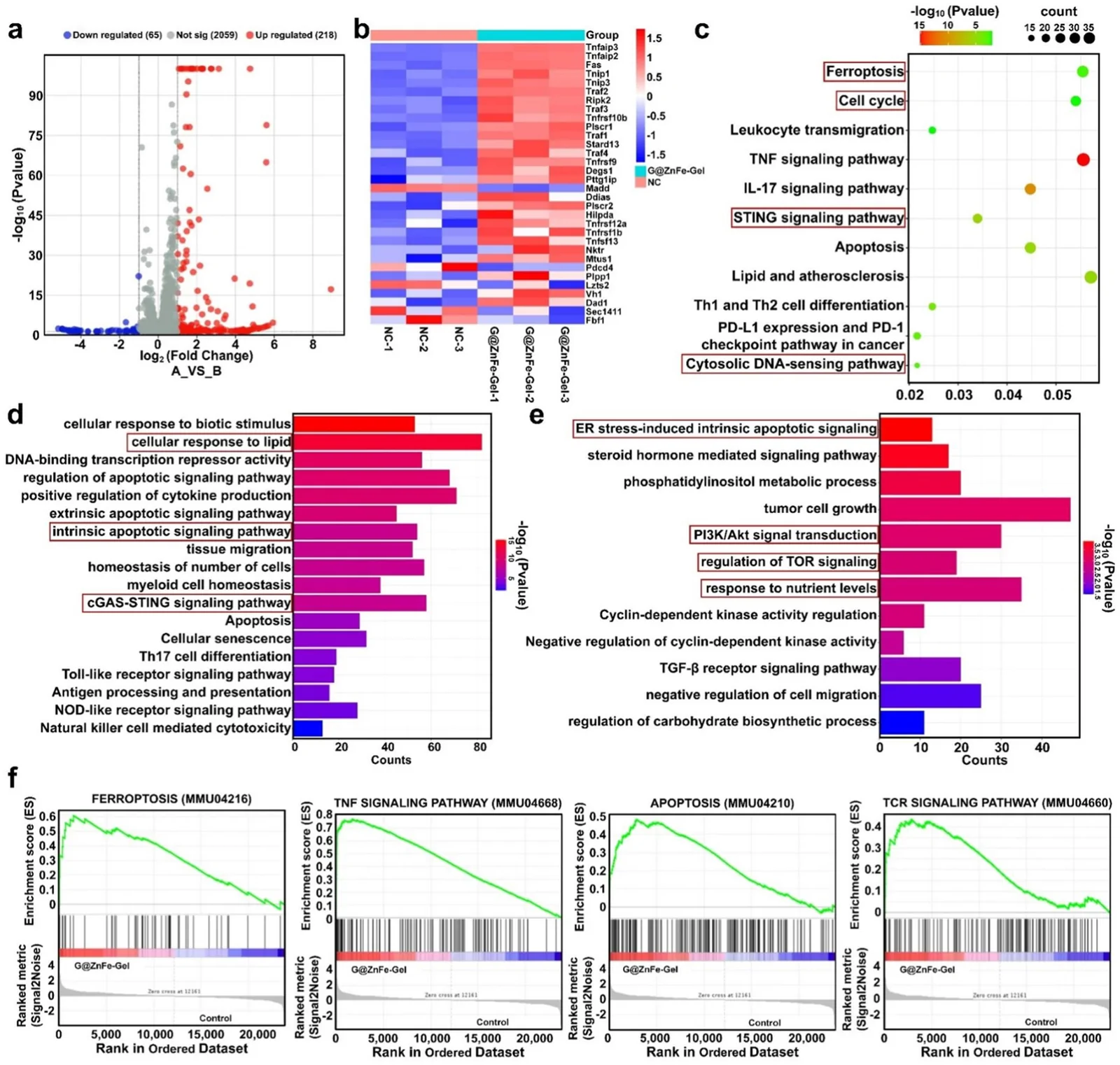
Transcriptomic profiling of PAN02 cells following G@ZnFe-Gel treatment. (a) Volcano plot showing differentially expressed genes (DEGs) between the G@ZnFe-Gel-treated group and the negative control (NC). Red dots indicate significantly upregulated genes (n = 218), and blue dots indicate significantly downregulated genes (n = 65). Screening criteria: |log_2_FC| > 1.5 and *P* < 0.05. (b) Heatmap of DEGs, with rows representing genes and columns representing biological replicates from the NC and G@ZnFe-Gel-treated groups. (c) KEGG pathway enrichment bubble plot of significantly enriched pathways following G@ZnFe-Gel treatment. Bubble size represents the number of enriched genes, and color intensity indicates statistical significance. (d-e) Gene Ontology (GO) biological process enrichment analysis. Bar length represents the number of enriched genes, and color intensity reflects enrichment significance. (f) Gene set enrichment analysis (GSEA) showing significant positive enrichment of ferroptosis, TNF signaling, apoptosis, and T-cell receptor signaling pathways in the G@ZnFe-Gel-treated group. NES, normalized enrichment score. (For interpretation of the references to color in this figure legend, the reader is referred to the Web version of this article.)

To assign functional significance to the differentially expressed genes (DEGs), KEGG pathway enrichment analysis was performed. As shown in Fig. 5c, these genes were prominently enriched in pathways associated with “ferroptosis”, “apoptosis”, “cGAS-STING signaling pathway”, and “cytosolic DNA sensing”. This transcriptional signature is highly consistent with the material design strategy of this study, which centers on Fe-mediated ROS amplification and lipid peroxidation to couple immunogenic cell death with innate immune activation. At a systems level, these data demonstrate that G@ZnFe-Gel not only effectively triggers iron-dependent oxidative cell death programs, but also concomitantly activates DNA-sensing-related innate immune pathways, providing transcriptomic evidence for the proposed ferroptosis-cGAS–STING synergistic amplification mechanism.

GO biological process enrichment analysis further reinforced these findings. Compared with the control group, G@ZnFe-Gel-treated cells exhibited significant enrichment in the “Ferroptosis”, “Glutathione metabolism”, and “cGAS–STING signaling pathway” (Fig. 5d). The concurrent activation of ferroptosis and glutathione metabolism pathways reflects a pronounced elevation of intracellular oxidative stress driven by sustained H_2_O_2_ production *via* GOD-catalyzed glucose oxidation and Fe^3+^-mediated Fenton-like reactions within the bimetallic MOF nanozyme framework. This oxidative burden leads to a systemic collapse of the GSH/GPX4-centered antioxidant defense network, thereby providing a molecular basis for irreversible lipid peroxidation accumulation and execution of ferroptosis. Meanwhile, the prominent enrichment of the cGAS-STING pathway aligns well with the mechanistic hypothesis that ferroptosis-associated mitochondrial DNA release, together with Zn^2+^-facilitated signal amplification, enhances cytosolic DNA sensing and innate immune activation, highlighting the immunological design logic of the bimetallic nanozyme system.

To experimentally validate key nodes within these pathways, representative ferroptosis-related genes (LPCAT1, NRF2, FTH1, and ACSL4, Fig. S17a-d) and STING-associated genes (IFIT1 and IFIT3, Fig. S17e and f) were further examined by qRT-PCR. The expression trends of these genes were highly consistent with the RNA-seq results, confirming that G@ZnFe-Gel transcriptionally reinforces ferroptosis driven by ROS amplification and lipid peroxidation, while simultaneously activating the STING-IFN-associated innate immune signaling axis. GO biological process enrichment analysis provided additional insight into the systemic suppression of tumor stress-adaptation programs by G@ZnFe-Gel. Pathways associated with endoplasmic reticulum stress-related intrinsic apoptosis and multiple metabolic reprogramming programs were significantly downregulated in the treatment group (Fig. 5e). These changes indicate that G@ZnFe-Gel not only exerts sustained oxidative and ferro-ptotic stress but also suppresses key pro-survival and compensatory metabolic adaptations that pancreatic cancer cells exploit within fibrotic and nutrient-restricted microenvironments. By disabling these buffering pathways, G@ZnFe-Gel drives tumor cells from a reversible adaptive state toward an irreversible programmed cell death outcome.

Notably, GSEA further revealed significant positive enrichment of the ferroptosis, tumor necrosis factor (TNF) signaling, apoptosis, and T-cell receptor (TCR) signaling pathways (Fig. 5f). Enrichment of the ferroptosis pathway confirms its direct role in driving tumor cell death through iron overload, lipid peroxidation, and ROS generation. Activation of the apoptosis pathway provides a complementary mode of regulated cell death, while enrichment of the TNF signaling pathway indicates the establishment of a pro-inflammatory tumor milieu conducive to immune activation [36]. Importantly, the positive enrichment of the TCR signaling gene set reflects treatment-associated transcriptional changes in PAN02 tumor cells. Complementary *in vivo* analyses showed increased intratumoral CD4^+^ and CD8^+^ T-cell infiltration and elevated IFN-γ expression (Fig. 7d, e, i, and j), supporting enhanced local immune engagement following G@ZnFe-Gel treatment.

Taken together, Integrated RNA-seq and qRT-PCR analyses demonstrate that G@ZnFe-Gel profoundly remodels the transcriptional landscape of pancreatic cancer cells in alignment with its structural and functional design. By activating ferroptosis-associated metabolic pathways and the cGAS-STING innate immune axis while suppressing tumor survival and metabolic adaptation programs, G@ZnFe-Gel establishes a molecular basis for transitioning from ferroptosis-driven tumor cell elimination to sustained immune-mediated tumor control. This mechanism is particularly suited to reprogramming dense and immunosuppressive pancreatic tumor microenvironments.

### 2.6 *In vivo* evaluation of antitumor efficacy and tumor growth inhibition

To systematically evaluate the *in vivo* therapeutic efficacy of G@ZnFe-Gel against pancreatic cancer, a subcutaneous PAN02 tumor model was established in mice, followed by a 20-day course of intratumoral treatment (Fig. 6a). At the experimental endpoint, tumors from different treatment groups exhibited pronounced macroscopic differences (Fig. 6b). Tumors in Control and blank hydrogel (FS-Gel) groups were large and compact, indicating that the carrier alone exerted negligible antitumor activity. In contrast, Zn-Gel group showed a modest reduction in tumor size, suggesting that Zn^2+^ alone may provide limited immunostimulatory effects but is insufficient for robust tumor suppression. Further tumor shrinkage was observed in ZnFe-Gel group, indicating that the introduction of Fe^3+^ and ferroptosis-related mechanisms substantially enhanced therapeutic efficacy. Notably, tumors in G@ZnFe-Gel group were the smallest, with some specimens displaying extensive fragmentation or necrotic features, highlighting the pronounced antitumor synergy arising from the cascade cooperation between GOD and dual-metal nanozyme.

**Fig. 6:**
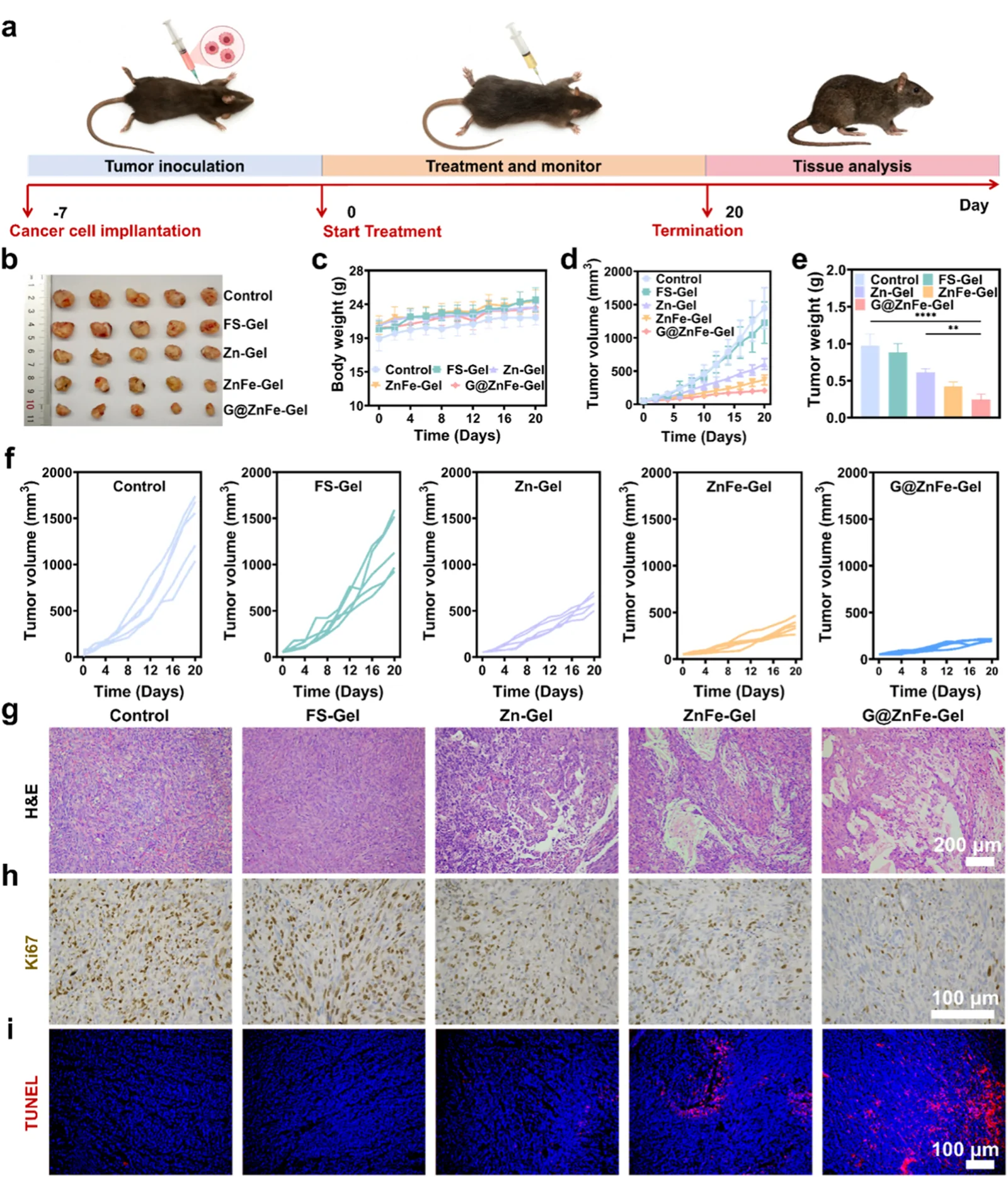
*In vivo* antitumor efficacy and tumor growth inhibition of G@ZnFe-Gel. (a) Schematic overview of the treatment regimen and experimental timeline in a mouse model of subcutaneous pancreatic cancer. (b) Representative photographs of excised tumors from different treatment groups at the experimental endpoint. (c) Body weight changes of mice during the treatment period. (d) Tumor volume growth curves of mice receiving different treatments. (e) Quantitative analysis of tumor weights at the experimental endpoint. (f) Individual tumor growth trajectories for mice in each treatment group (n = 5). (g) H&E staining of tumor tissue sections. (h) Immunohistochemical staining of Ki67 to assess tumor cell proliferation. (i) TUNEL staining of tumor sections to evaluate apoptosis. *P < 0.05,**P < 0.01,***P < 0.001,****P < 0.0001.

**Fig. 7:**
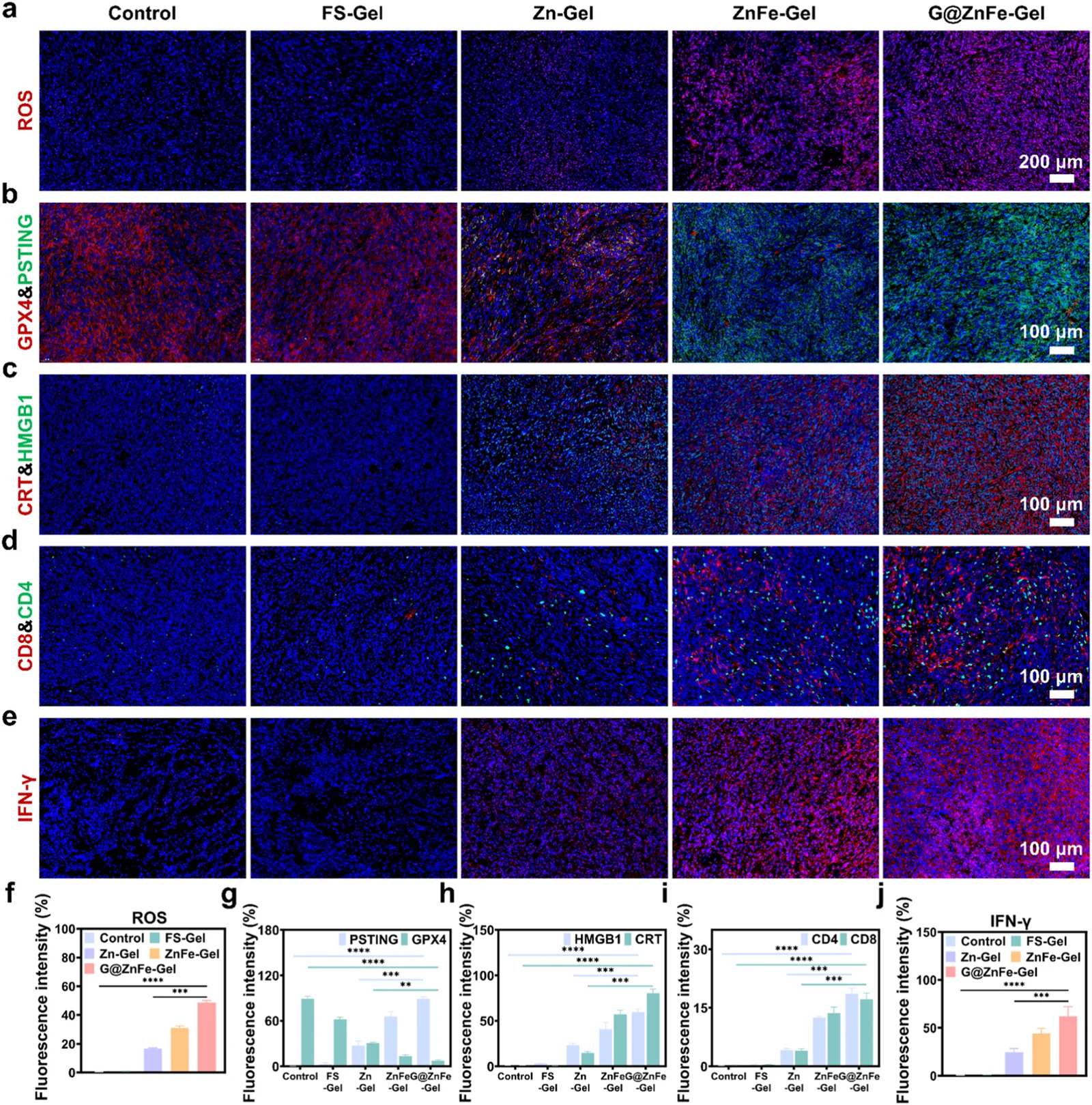
Mechanistic investigation of G@ZnFe-Gel–mediated antitumor immune activation. (a) Immunofluorescence images of reactive oxygen species (ROS) levels in tumor tissues stained with Cell ROX™ Deep Red (red), with nuclei counterstained by DAPI (blue). (b) Immunofluorescence co-staining of GPX4 (red) and STING (green) in tumor tissues. (c) Immunofluorescence co-staining of immunogenic cell death (ICD) markers calreticulin (CRT, red) and HMGB1 (green) in tumor tissues. (d) Immunofluorescence staining of infiltrating CD8^+^ cytotoxic T cells (red) and CD4^+^ helper T cells (green) in tumor tissues. (e) Immunofluorescence staining of IFN-γ expression in tumor tissues. (f) Quantitative analysis of ROS fluorescence intensity corresponding to panel (a) (n = 3). (g) Quantitative analysis of GPX4 and STING fluorescence signals corresponding to panel (b) (n = 3). (h) Quantitative analysis of CRT and HMGB1 fluorescence signals corresponding to panel (c) (n = 3). (i) Quantitative analysis of CD8^+^ and CD4^+^ T-cell infiltration corresponding to panel (d) (n = 3). (j) Quantitative analysis of IFN-γ fluorescence intensity corresponding to panel (e) (n = 3). *P < 0.05,**P < 0.01,***P < 0.001,****P < 0.0001. (For interpretation of the references to color in this figure legend, the reader is referred to the Web version of this article.)

Longitudinal monitoring of tumor growth curves further corroborated these observations (Fig. 6d). Tumors in the Control and FS-Gel groups exhibited rapid and continuous expansion with typical exponential growth kinetics, confirming the lack of growth inhibition by the hydrogel matrix alone. It is worth noting that FS-Gel relies primarily on ionic crosslinking between SA and Ca^2+^ enriched in the tumor microenvironment, resulting in a relatively loose network with limited capacity for sustained retention and localized therapeutic action. Compared with FS-Gel, Zn-Gel group showed a partial deceleration in tumor growth, yet tumors continued to expand overall, underscoring the limited antitumor efficacy of Zn^2+^ alone. Upon incorporation of Fe^3+^, ZnFe-Gel induced a pronounced attenuation of tumor growth, as evidenced by a marked flattening of the tumor growth curve, consistent with ferroptosis-mediated amplification of therapeutic effects.

Strikingly, G@ZnFe-Gel nearly completely arrested tumor progression throughout the entire treatment period, with tumor volumes remaining at consistently low levels, demonstrating the most potent *in vivo* antitumor activity among all groups. This superior efficacy can be attributed not only to the ferroptosis-immune synergistic mechanism but also to the material's rapid and robust in situ gelation behavior. In G@ZnFe-Gel, Zn^2+^/Fe^3+^ ions and GOD participate in therapeutic reactions while simultaneously reinforcing the hydrogel network through multivalent ionic crosslinking and physical confinement. Together with Ca^2+^-mediated alginate crosslinking, these interactions substantially increase network density, mechanical integrity, and structural stability, thereby enabling efficient intratumoral fixation and controlled release of therapeutic components. Such material-level reinforcement provides critical support for overcoming the dense stromal barrier characteristic of pancreatic tumors.

Quantitative analysis of tumor weights at the endpoint (Fig. 6e) was fully consistent with the tumor volume measurements. No significant difference was observed between Control and FS-Gel groups, whereas G@ZnFe-Gel group exhibited the most pronounced reduction in tumor mass (***P < 0.0001), representing an additional about 40% decrease compared with ZnFe-Gel group. These results clearly demonstrate that incorporation of GOD markedly enhances overall antitumor efficacy by amplifying the cascade effects of ferroptosis induction and cGAS-STING-mediated immune activation. Furthermore, individual tumor growth trajectories (Fig. 6f) revealed substantial inter-animal variability in Control and FS-Gel groups, reflecting the intrinsic heterogeneity of pancreatic cancer. This variability was reduced in Zn-Gel and ZnFe-Gel groups, while all animals treated with G@ZnFe-Gel displayed uniformly suppressed and stable tumor growth, underscoring the robustness, consistency, and reproducibility of this therapeutic strategy *in vivo*.

To further substantiate the antitumor effects at the tissue level, excised tumors were subjected to histopathological and immunohistochemical analyses. Hematoxylin and eosin (H&E) staining (Fig. 6g) revealed densely packed tumor cells with intact nuclear-cytoplasmic architecture and only sporadic focal necrosis in Control and FS-Gel groups. The Zn-Gel group exhibited localized regions of tumor cell necrosis, whereas the ZnFe-Gel group displayed more extensive necrotic areas accompanied by disrupted cellular morphology and fragmentation. In sharp contrast, tumors treated with G@ZnFe-Gel showed widespread vacuolization and diffuse necrosis, with a dramatic reduction in tumor cell density, indicating severe tumor tissue destruction *in vivo*.

Ki67 immunostaining was employed to assess tumor cell proliferative activity [37]. Abundant Ki67-positive nuclei were detected in Control group, consistent with a highly proliferative tumor phenotype. Little change was observed in FS-Gel group, whereas a progressive decrease in Ki67-positive cells was evident in Zn-Gel and ZnFe-Gel groups, reflecting graded suppression of proliferation. Remarkably, Ki67-positive signals were nearly absent in G@ZnFe-Gel group, indicating profound inhibition of tumor cell proliferation. Terminal deoxynucleotidyl transferase dUTP nick-end labeling (TUNEL) staining further revealed the strongest apoptotic or necrotic signals in the G@ZnFe-Gel group (Fig. S18), significantly exceeding those observed in all other groups, demonstrating extensive tumor cell death *in vivo*.

Collectively, these pathological and quantitative analyses indicate that G@ZnFe-Gel drives pronounced tumor tissue destruction, effectively suppresses proliferation, and triggers extensive programmed cell death through the synergistic induction of ferroptosis and activation of the cGAS-STING pathway. This coordinated mechanism underlies the robust and durable antitumor efficacy of G@ZnFe-Gel *in vivo*.

### 2.7 *In vivo* investigation of antitumor immune mechanisms

To elucidate the immunoregulatory mechanisms underlying the in situ immunotherapeutic efficacy of G@ZnFe-Gel in pancreatic cancer, a comprehensive *in vivo* analysis was performed using immunofluorescence and immunohistochemistry. Multiple interconnected processes were systematically examined, including oxidative stress induction, ferroptosis activation, cGAS-STING-mediated innate immune signaling, immunogenic cell death (ICD) initiation, and remodeling of the tumor immune microenvironment (TIME). ROS fluorescence staining revealed that tumors treated with G@ZnFe-Gel exhibited the most intense red fluorescence signals among all groups (Fig. 7a and f), indicating robust intratumoral ROS generation. This pronounced oxidative stress was likely attributed to sustained H_2_O_2_ production driven by GOD-mediated glucose oxidation, followed by metal ion-catalyzed Fenton-like reactions that convert H_2_O_2_ into highly reactive **·**OH. Consistently, both lipid peroxidation (LPO) levels and malondialdehyde (MDA) content were significantly elevated in G@ZnFe-Gel–treated tumors (Fig. S19), confirming extensive oxidative lipid damage *in vivo*.

GPX4 immunofluorescence staining provided additional evidence of ferroptosis-associated changes. As shown in Fig. 7b and g, G@ZnFe-Gel treatment led to a marked reduction in GPX4 fluorescence intensity compared with all control groups, indicating effective suppression of the GPX4-dependent antioxidant defense and robust induction of ferroptosis. Given the close mechanistic coupling between ferroptosis and innate immune sensing, we next examined activation of the cGAS-STING pathway. Immunohistochemical analysis demonstrated that both STING expression and its downstream effector IFN-β were significantly upregulated in tumors treated with G@ZnFe-Gel (Fig. S20). These results suggest that ferroptosis-associated release of damage-associated molecular patterns (DAMPs), together with Zn^2+^ signaling, synergistically activates the cGAS–STING axis, thereby providing a critical driving force for subsequent immune amplification.

Ferroptosis is increasingly recognized as a highly immunogenic form of regulated cell death capable of promoting tumor antigen exposure and transfer [38]. Accordingly, immunofluorescence staining of two canonical ICD markers, calreticulin (CRT) and high-mobility group box 1 (HMGB1), was conducted. As shown in Fig. 7c and h, G@ZnFe-Gel treatment elicited the strongest CRT surface exposure and HMGB1 release among all groups, demonstrating potent ICD induction. CRT exposure facilitates dendritic cell (DC) recognition and phagocytosis of tumor antigens, whereas HMGB1 release promotes DC maturation through TLR4 signaling [39]. These findings confirmed that G@ZnFe-Gel induced ferroptosis was highly immunogenic and effectively enhanced DC-mediated antigen uptake and presentation.

Tumor-associated macrophages (TAMs), which predominantly exhibit a pro-tumorigenic M2 phenotype in pancreatic cancer, are a major contributor to immunosuppression within the TIME [40]. Immunofluorescence staining of CD86 (M1 marker) and CD163 (M2 marker) revealed that G@ZnFe-Gel treatment significantly decreased CD163 expression while markedly increasing CD86 signals (Fig. S21 and S22). This phenotype shift indicated effective repolarization of TAMs from an immunosuppressive M2 state toward a pro-inflammatory, tumoricidal M1 phenotype, thereby alleviating immune suppression and creating a permissive microenvironment for T-cell infiltration.

At the level of adaptive immunity [41], tumor-infiltrating T lymphocytes were further analyzed. Immunofluorescence staining demonstrated that G@ZnFe-Gel treatment significantly enhanced the infiltration of CD8^+^ cytotoxic T lymphocytes (CTLs) and CD4^+^ helper T cells compared with other treatment groups (Fig. 7d and i). This increased T-cell recruitment was likely driven by the combined effects of ferroptosis-induced ICD and cGAS-STING-mediated innate immune activation, which together promoted efficient priming and amplification of adaptive immune responses [42]. To assess the functional status of infiltrating T cells, IFN-γ expression was evaluated by immunohistochemistry. As shown in Fig. 7e and j, G@ZnFe-Gel treatment resulted in the strongest IFN-γ signals, reaching approximately 60-fold higher levels than those in control tumors. As a hallmark effector cytokine of Th1-type immune responses, IFN-γ not only directly inhibits tumor cell proliferation but also enhances antigen presentation and sustains M1 macrophage polarization [43], thereby reinforcing antitumor immunity within the TIME.

Overall, these results confirm that G@ZnFe-Gel orchestrates a robust *in vivo* immune cascade by amplifying ROS production, inducing ferroptosis, and activating the cGAS-STING signaling pathway. This integrated process establishes a coordinated innate-adaptive immune amplification loop, characterized by TAM reprogramming, enhanced CTL infiltration, and sustained effector cytokine production. Through these mechanisms, G@ZnFe-Gel effectively reshapes the immunosuppressive pancreatic cancer microenvironment and elicits a potent and durable antitumor immune response.

### 2.8 *In vivo* assessment of biosafety and systemic biocompatibility

Given the pronounced antitumor efficacy of G@ZnFe-Gel observed in the subcutaneous pancreatic cancer model, its *in vivo* biosafety and systemic biocompatibility were further systematically evaluated, which is a critical prerequisite for potential clinical translation. Dynamic monitoring of body weight during the treatment period revealed that mice in all experimental groups maintained stable body weights without significant loss or abnormal fluctuations (Fig. 6c), indicating that the administered formulations, including G@ZnFe-Gel, did not induce overt systemic toxicity at the tested dose. At the experimental endpoint, serum biochemical analyses were performed to assess potential hepatic and renal toxicity. Levels of aspartate aminotransferase (AST) and alanine aminotransferase (ALT), two key indicators of hepatocellular integrity, remained within the normal physiological range across all treatment groups (Fig. 8a and b), suggesting the absence of detectable liver injury. Meanwhile, biomarkers reflecting renal function, including creatinine (CRE) and blood urea nitrogen (BUN), showed no significant differences among groups (Fig. 8c and d), indicating that the treatments did not elicit measurable nephrotoxicity. Collectively, these results demonstrate that the local retention and sustained-release characteristics of the hydrogel-based delivery system effectively mitigate the liver and kidney toxicity commonly associated with systemic administration.

**Fig. 8:**
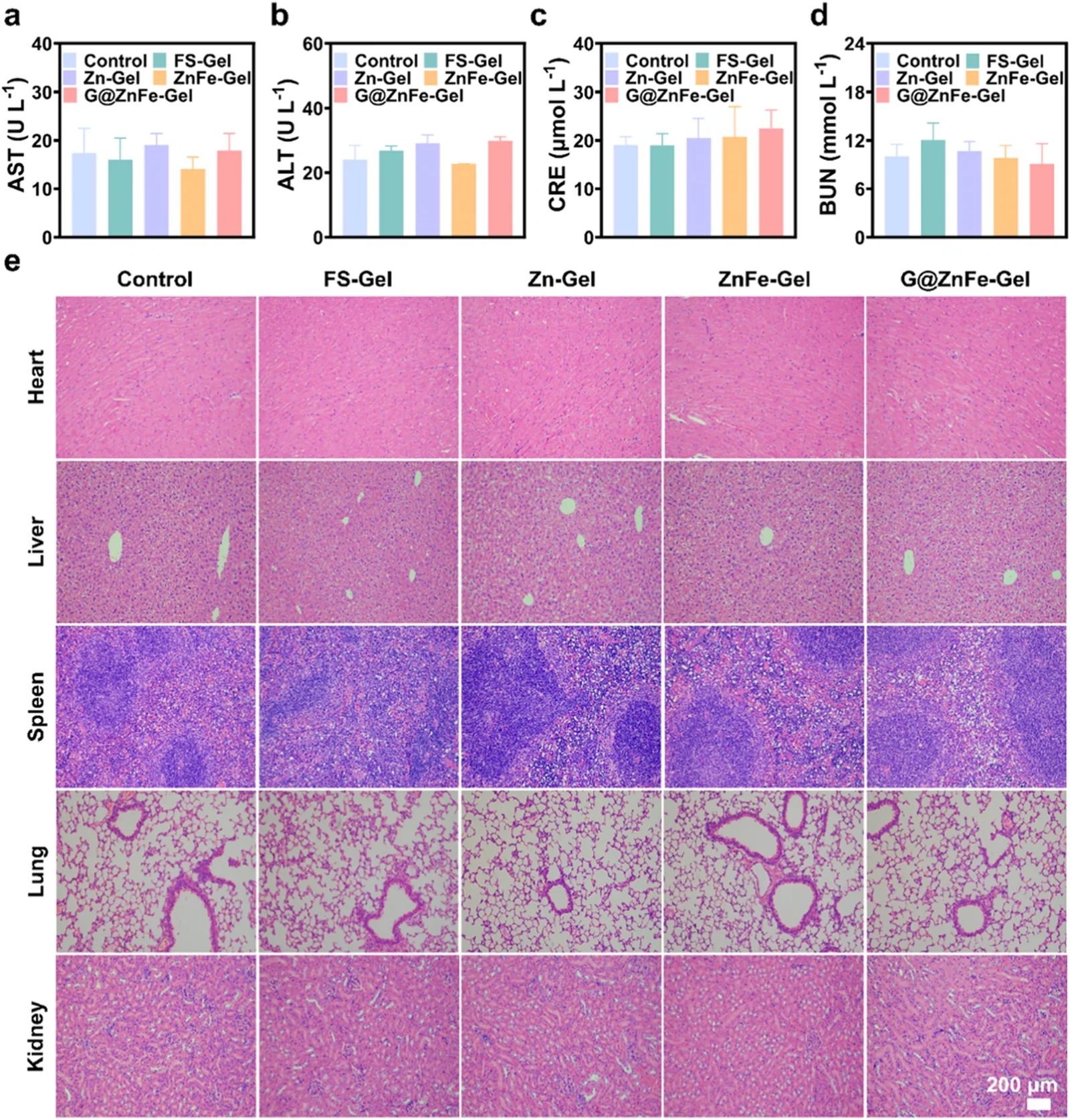
*In vivo* biosafety evaluation of G@ZnFe-Gel. (a-d) Serum biochemical analysis of (a) alanine aminotransferase (ALT), (b) aspartate aminotransferase (AST), (c) blood urea nitrogen (BUN), and (d) creatinine (CRE) levels in mice following various treatments (n = 5). (e) Representative H&E-stained histological images of major organs (heart, liver, spleen, lung, and kidney) collected from each treatment group.

To further substantiate the *in vivo* safety profile of G@ZnFe-Gel, histopathological examinations of major organs, including the heart, liver, spleen, lungs, and kidneys—were conducted at the treatment endpoint using H&E staining (Fig. 8e). All examined organs exhibited intact and normal tissue architectures, with no evidence of inflammatory infiltration, necrosis, structural disruption, or abnormal material deposition across treatment groups. These histological findings further confirmed that G@ZnFe-Gel did not induce discernible pathological damage to distal normal tissues.

Altogether, these results suggest that G@ZnFe-Gel combines potent antitumor efficacy with excellent systemic safety, supporting its promise as a clinically relevant in situ immunotherapeutic strategy for pancreatic cancer.

## 3 Conclusion

In conclusion, we developed a microenvironment-responsive injectable marine polysaccharide hydrogel system reinforced by a Zn/Fe bimetallic MOF nanozyme to address the dual barriers of dense stroma and immune suppression in pancreatic cancer. By integrating thermally triggered gelation, Ca^2+^-mediated ionic crosslinking, and nanozyme-induced network reinforcement, the hydrogel rapidly forms a stable intratumoral depot, ensuring sustained local retention within the stromal-rich tumor microenvironment. By mechanistically coupling ferroptosis with cGAS-STING activation, the platform transforms localized redox perturbation into sustained immune engagement, ultimately re-establishing immune surveillance within the pancreatic tumor microenvironment. Further studies are needed to assess long-term Zn/Fe biodistribution, metabolism, clearance, and safety, evaluate immune memory and protection against tumor rechallenge, and validate efficacy in more representative models before clinical translation.

## CRediT authorship contribution statement

**Yihao Zhan:** Writing – original draft, Software, Methodology, Conceptualization. **Chuanqin Shi:** Writing – original draft, Software, Methodology, Formal analysis, Conceptualization. **Jiqing Dong:** Formal analysis, Data curation. **Shihua Zhao:** Formal analysis, Data curation. **Xintao Gao:** Formal analysis, Data curation. **Junyao Li:** Formal analysis, Data curation. **Xiangyan Chen:** Writing – review & editing, Supervision, Resources, Funding acquisition, Conceptualization. **Yantao Li:** Writing – review & editing, Supervision, Resources, Funding acquisition, Conceptualization.

## Declaration of competing interests

The authors declare that they have no known competing financial interests or personal relationships that could have appeared to influence the work reported in this paper.

## Data Availability

Data will be made available on request.
