# Supplementary material for: Microenvironment-responsive injectable marine polysaccharide hydrogel empowered by bimetallic MOF nanozymes for in situ pancreatic cancer immunotherapy via ferroptosis-mediated cGAS-STING activation
Source: Mater Today Bio. 2026 Oct 9;41:103735. doi: 10.1016/j.mtbio.2026.103735 (PMC13679371; doi:10.1016/j.mtbio.2026.103735)
Supplement: Supplementary file 1 [file mmc1.docx]

**Supporting Information**

**Microenvironment-Responsive Injectable Marine Polysaccharide Hydrogel Empowered by Bimetallic MOF Nanozymes for In Situ Pancreatic Cancer Immunotherapy *via* Ferroptosis-Mediated cGAS-STING Activation**

Yihao Zhan^a,1^, Chuanqin Shi^b,1^, Jiqing Dong^a^, Shihua Zhao^b^, Xintao Gao^a^, Junyao Li^a^, Xiangyan Chen^a,*^, Yantao Li^a,**^

^a^ State Key Laboratory of Advanced Optical Polymer and Manufacturing Technology, Qingdao Key Laboratory of Biomacromolecular Drug Discovery and Development, College of Chemical Engineering, Qingdao University of Science and Technology, Qingdao, 266042, China

^b^ Center of Translational Medicine, Zibo Central Hospital Affiliated to Binzhou Medical University, Zibo, 255020, China.

* Corresponding author.

** Corresponding author.

^1^ These authors contributed equally.

E-mail: cxy@qust.edu.cn (X.Y. Chen); liyantao@qust.edu.cn (Y.T. Li)

# Experimental Section

**Chemicals and Reagents**

Zinc nitrate hexahydrate (Zn(NO_3_)_2_·6H_2_O, 99%) was purchased from Xilong Chemical Co., Ltd. (Beijing, China). Iron(III) acetylacetonate (Fe(acac)_3_, 98%), 2-methylimidazole (2-MIM, 98%), and calcium chloride (CaCl_2_, 98%) were purchased from Aladdin Biochemical Technology Co., Ltd. (Shanghai, China). Sodium alginate (SA, viscosity 60 mPa·s, Mw = 70 kDa, M/G = 6/4) were purchased from Qingdao gather great ocean algae industry group Co. Ltd. (Qingdao, China). Glucose oxidase (GOD) and D-glucose were obtained by Macklin Biochemical Co., Ltd. (Shanghai, China). Pluronic F127 was purchased from Solarbio Science & Technology Co., Ltd. (Beijing, China). Methanol, 3,3′,5,5′-tetramethylbenzidine (TMB, 98%), and dimethyl sulfoxide (DMSO) were obtained from Sinopharm Chemical Reagent Co., Ltd. (Shanghai, China). All chemicals were used as received without further purification. Ultrapure water with a resistivity of 18.2 MΩ·cm (Millipore Milli-Q system) was used throughout all experiments.

**Preparation of Zn/Fe Bimetallic MOF Nanozymes and Glucose Oxidase-Loaded Nanoassemblies**

***Preparation of Zn-ZIF NPs.*** Zinc nitrate hexahydrate (0.167 mmol) was dissolved in 2 mL of methanol. Separately, 2-methylimidazole (2-MI, 1.34 mmol) was dissolved in 2 mL of methanol. Under continuous magnetic stirring, the zinc precursor solution was slowly added dropwise into the 2-MI solution. The reaction mixture was stirred for an additional 1 h at room temperature. After completion, the resulting precipitate was collected by centrifugation at 10 000 rpm for 5 min and sequentially washed with 4 mL methanol (once), 2 mL methanol/deionized water mixture (once), and 4 mL deionized water (once). The obtained Zn-ZIF NPs were collected and stored for subsequent use.

***Preparation of ZnFe-ZIF NPs.*** Zinc nitrate hexahydrate (0.167 mmol) was dissolved in 1 mL of methanol, while iron (III) acetylacetonate (0.1 mmol) was dissolved in another 1 mL of methanol. The two metal precursor solutions were mixed thoroughly to form a homogeneous solution. Separately, 2-methylimidazole (1.34 mmol) was dissolved in 2 mL of methanol. Under stirring, the mixed metal salt solution was slowly added dropwise into the 2-MI methanolic solution, followed by continuous stirring for 1 h. The resulting suspension was centrifuged at 10000 rpm for 5 min to collect the precipitate, which was subsequently washed once with 4 mL methanol, once with 2 mL methanol/deionized water mixture, and once with 4 mL deionized water. The purified ZnFe-ZIF NPs were collected for further use.

***Preparation of G@ZnFe-ZIF NPs.*** Glucose oxidase (GOD, 10 mg) was dissolved in 2 mL of deionized water under gentle stirring for 1 h to ensure complete dissolution. The washed ZnFe-ZIF NPs were dispersed in a mixed solvent consisting of 0.6 mL methanol and 1.4 mL deionized water. Under stirring, the ZnFe-ZIF NPs dispersion was slowly added dropwise into the GOD aqueous solution, followed by further incubation with stirring for 2 h to facilitate enzyme loading. After completion, the resulting G@ZnFe-ZIF NPs were collected by centrifugation and washed once with deionized water. The final products were collected and stored for subsequent experiments.

***In Vitro* Evaluation of Enzymatic Cascade Reactions**

First, 3,3′,5,5′-tetramethylbenzidine (TMB, 1.92 mg) was dissolved in 1 mL of dimethyl sulfoxide (DMSO) to prepare a TMB stock solution, which was protected from light and gently shaken until complete dissolution. Subsequently, 500 μL of HAc–NaAc buffer (pH = 5.6) was mixed sequentially with 100 μL of the TMB stock solution, 200 μL of glucose solution (10 mM), and 100 μL of deionized water. The reaction was initiated by adding 100 μL of the sample solution, followed by incubation at room temperature under dark conditions to allow color development. The absorbance spectra of the reaction system were recorded in the wavelength range of 500–800 nm using a UV–vis spectrophotometer. The characteristic absorbance at 652 nm was monitored over time (5-120 min) to obtain kinetic profiles, thereby evaluating the efficiency of the enzymatic cascade reaction involving GOD-mediated H_2_O_2_ generation and the subsequent Fe^3+^-mediated Fenton-like catalytic process.

***Evaluation of GOD Enzymatic Activity and Stability***

Free GOD and G@ZnFe-ZIF containing equivalent amounts of GOD were incubated with glucose (1 mg mL^-1^) in PBS (pH 7.4) at 37°C. Samples were collected at 0, 2, 4, and 6 h, heated in boiling water for 15 min to terminate the reaction, and the residual glucose concentration was determined using a commercial HK/G6PDH-based glucose assay at 340 nm.

For stability evaluation, free GOD and G@ZnFe-ZIF containing equivalent GOD amounts (2 μg) were incubated in PBS containing 10% (v/v) FBS at 37°C for 0, 12, 24, and 48 h. After incubation, glucose was added to 1 mg mL^-1^ and allowed to react for 4 h. Residual glucose was quantified as described above, and the activity at 0 h for each formulation was normalized to 100% to calculate the relative residual activity.

**Preparation of Pluronic F127/Alginate Hydrogels and Nanozyme-Reinforced Composite Hydrogels**

Pluronic F127 (800 mg) and SA (40 mg) were dissolved in 4 mL of deionized water at 4°C under gentle stirring until a homogeneous solution was obtained. The mixture was allowed to stand for 2 h at 4°C to remove entrapped air bubbles. Subsequently, the solution was poured into a predefined mold and incubated at 37°C for approximately 3 min, during which thermos-responsive physical gelation of Pluronic F127 occurred, yielding the FS-Gel.

To prepare the nanozyme-reinforced hydrogel, Pluronic F127 (800 mg) and sodium alginate (40 mg) were dissolved in 4 mL of Zn-ZIF NPs, ZnFe-ZIF NPs, or G@ZnFe-ZIF NPs dispersion at 4°C under continuous stirring until complete homogenization. The resulting mixtures were allowed to stand at 4°C for 2 h to eliminate air bubbles. The degassed solutions were then transferred into molds and incubated at 37°C for approximately 3 min to induce thermos-responsive gelation of Pluronic F127, yielding the Zn-Gel, ZnFe-Gel, and G@ZnFe-Gel, respectively.

**Rheological Properties, Injectability, and Sol-Gel Phase Transition**

***Rheological Characterization of G@ZnFe-Gel.*** Prior to testing, the samples were thoroughly homogenized and degassed to eliminate air bubbles. Rheological measurements were performed using a rheometer equipped with a 20 mm diameter roughened parallel-plate geometry, with the temperature maintained at 37°C.

Amplitude sweep tests were conducted at a fixed frequency of 1 Hz over a strain range of 0.01-1000% (logarithmic increment) to determine the linear viscoelastic region (LVR) and yield point. Frequency sweep measurements were subsequently carried out over a range of 0–20 Hz to evaluate the frequency-dependent behavior of the storage modulus (G′) and loss modulus (G″). Time sweep tests were performed within the LVR at a low strain (γ = 0.2%) for 0–600 s to monitor the temporal evolution of viscoelastic moduli, which was used to assess gel stability or in situ gelation kinetics. Each experiment was conducted in triplicate.

***Injectability of G@ZnFe-Gel.*** The G@ZnFe-Gel was loaded into a 5 mL syringe and extruded at a constant plunger speed of 50 mm min^-1^ into a CaCl_2_ solution (2 mM) maintained at 37°C. The injectability and shape fidelity of the hydrogel during extrusion and post-injection were visually observed and documented by photography.

***Sol-Gel Phase Transition Behavior of G@ZnFe-Gel.*** The G@ZnFe-Gel precursor solution was fully dissolved in an ice bath and equilibrated at 4°C prior to aliquoting into capped glass vials. The sol–gel transition behavior was evaluated using the vial inversion method. Briefly, at 4°C, the vial was tilted or inverted; samples that flowed within 10 s were defined as being in the sol state. The vials were then transferred to a temperature-controlled environment at ≥30 °C (typically 37°C) for approximately 3 min and inverted again. Samples without observable flow were classified as thermally induced gels.

For ion-triggered gelation, 100 μL of CaCl_2_ solution (2 mM) was added dropwise to the same sample, gently mixed, and allowed to stand at room temperature for 1 min. The vial was then inverted, and samples that did not flow were considered to have formed Ca^2+^-induced gels. Temperature, incubation time, and representative images or videos were recorded throughout the process. All experiments were performed in triplicate to ensure reproducibility.

***In Vivo* Retention and Degradation Behavior of G@ZnFe-Gel**

To evaluate *in vivo* retention and degradation, G@ZnFe-Gel was labeled with the near-infrared fluorescent dye IR-780. The labeled hydrogel was administered via in situ subcutaneous injection, and fluorescence signals were monitored using a small-animal *in vivo* imaging system at predetermined time points (0 h, 12 h, 24 h, 2 d, 4 d, and 7 d). The temporal changes in fluorescence intensity were used to assess the *in vivo* retention and degradation behavior of the hydrogel.

**Hemolysis Assay of G@ZnFe-Gel**

The hemolytic activity of G@ZnFe-Gel was evaluated using freshly collected mouse whole blood obtained with ethical approval. Briefly, whole blood was centrifuged to remove plasma, and the red blood cells (RBCs) were repeatedly washed with sterile phosphate-buffered saline (PBS) until the supernatant became colorless. The washed RBCs were then resuspended in PBS to obtain an approximately 2% (v/v) erythrocyte suspension. Sterilized G@ZnFe-Gel was prepared as an extract solution at a concentration of 0.2 g mL^-1^ and mixed with the RBC suspension at an equal volume ratio. The mixtures were incubated at 37°C for 1 h. PBS and 0.1% Triton X-100 were used as the negative and positive controls, respectively. After incubation, the samples were centrifuged at 1000 g for 5 min, and the absorbance of the collected supernatants was measured at 540-545 nm using a microplate reader. Sample blanks without RBCs were used to subtract background absorbance. The hemolysis ratio (%) was calculated according to the following equation:

$$\text{Hemolysis (\%)}=\frac{A_{s}-A_{n}}{A_{p}-A_{n}}\times100\%$$

Where As, An, and Ap represent the absorbance values of the sample, negative control, and positive control, respectively. Each group was tested in triplicate at minimum, and representative images of the supernatants were recorded to visually assess hemolytic behavior.

**Cytotoxicity Evaluation *In Vitro***

***MTT Assay for Cell Viability of PAN02 Cells Treated with Hydrogels*** Mouse pancreatic cancer cells (PAN02) were cultured in RPMI-1640 medium supplemented with 10% fetal bovine serum (FBS) and 1% penicillin–streptomycin at 37°C in a humidified atmosphere containing 5% CO_2_. Cells were seeded into 96-well plates at a density of 5 × 10^3^ cells per well and allowed to adhere overnight. Sterilized FS-Gel, Zn-Gel, ZnFe-Gel, and G@ZnFe-Gel were prepared at various concentrations (1-16 mg mL^-1^) and incubated with PAN02 cells (Cell Resource Center, Shanghai Institute of Life Sciences, Chinese Academy of Sciences). After 24 h of treatment, cell viability was evaluated using a standard MTT assay. Briefly, MTT solution was added to each well to a final concentration of 0.5 mg mL^-1^ and incubated for 4 h. The absorbance was then measured using a microplate reader. Cell viability was calculated according to the following equation:

Cell viability (%) = (OD_sample_ − OD_blank_) / (ODnegative _control_ − OD_blank_) × 100

***Live/dead cell staining.*** Mouse pancreatic cancer cells (PAN02) were cultured in RPMI-1640 medium supplemented with 10% fetal bovine serum (FBS) and 1% penicillin–streptomycin at 37°C in a humidified atmosphere containing 5% CO_2_. Cells were seeded into 96-well plates at a density of approximately 5 × 10^3^ cells per well and allowed to adhere overnight. The cells were then treated with sterilized FS-Gel, Zn-Gel, ZnFe-Gel, or G@ZnFe-Gel at concentrations ranging from 1 to 16 mg mL^-1^ for 24 h. After treatment, cells were stained with Calcein-AM (2 μM, Yeasen, 40747ES76) and propidium iodide (PI, 5 μg mL^-1^, Yeasen, C3901200) for 15 min according to the manufacturer’s instructions. Fluorescence images were acquired using a fluorescence microscope, and cell viability was subsequently quantified based on the ratio of live (Calcein-AM–positive) to dead (PI-positive) cells.

**Flow Cytometric Analysis of Apoptosis**

PAN02 cells were seeded into 6-well culture plates at a density of 1 × 10^5^ cells per well and allowed to adhere overnight. Cells were then treated with FS-Gel, Zn-Gel, ZnFe-Gel, or G@ZnFe-Gel at a final concentration of 12 mg mL^-1^ for 24 h. Following treatment, cells were harvested, washed twice with cold phosphate-buffered saline (PBS), and resuspended in binding buffer. Apoptotic cells were stained with Annexin V–FITC and propidium iodide (PI) according to the manufacturer’s protocol and incubated in the dark for 15 min at room temperature. The stained cells were subsequently analyzed by flow cytometry to quantify early and late apoptotic populations. Data are presented as mean ± standard deviation (SD), and statistical analyses were performed as described in the Statistical Analysis section.

**Detection of Intracellular Reactive Oxygen Species (ROS)**

Intracellular reactive oxygen species (ROS) generation in PAN02 cells was evaluated using the fluorescent probe 2′,7′-dichlorodihydrofluorescein diacetate (DCFH-DA; Beyotime). PAN02 cells in the logarithmic growth phase were seeded into black-walled, clear-bottom 96-well plates at a density of 5 × 10^3^ cells per well and allowed to adhere overnight. The culture medium was then replaced with fresh medium containing sterilized FS-Gel, Zn-Gel, ZnFe-Gel, or G@ZnFe-Gel at concentrations ranging from 1 to 16 mg mL^-1^, with PBS-treated cells serving as the blank control. An H_2_O_2_-treated group was included as a ROS-positive control to verify the responsiveness of the DCFH-DA assay. After 12 h of incubation, the medium was removed and cells were gently washed twice with pre-warmed phosphate-buffered saline (PBS). Cells were subsequently incubated with DCFH-DA (10 µM, diluted in serum-free medium) at 37°C in the dark for 30 min. Following incubation, excess probe was removed, and the cells were washed three times with PBS to eliminate residual dye. Bright-field and fluorescence images were acquired under identical imaging settings for all groups, and merged images were generated to confirm the spatial correspondence between adherent cells and intracellular DCF fluorescence. Fluorescence images were immediately acquired using a fluorescence microscope (Nikon Eclipse Ti2, Japan). Quantitative analysis was performed using ImageJ software by calculating the mean fluorescence intensity (MFI) from at least three randomly selected fields per well. All experiments were independently repeated three times.

**Western Blot Analysis**

PAN02 cells in the logarithmic growth phase were seeded into 6-well plates at a density of 5 × 10^5^ cells per well and allowed to adhere overnight. Cells were then treated with different formulations, including Control, FS-Gel, Zn-Gel, ZnFe-Gel, and G@ZnFe-Gel, with three biological replicates per group. After 6 h of treatment, the culture medium was removed, and cells were gently washed twice with ice-cold phosphate-buffered saline (PBS). Cells were lysed on ice using RIPA lysis buffer supplemented with phenylmethylsulfonyl fluoride (PMSF) (Beyotime, #P0013B) for 30 min with intermittent agitation to ensure complete lysis. The lysates were collected using a cell scraper and centrifuged at 12,000 rpm for 15 min at 4°C. The supernatants were harvested as total protein extracts. Protein concentrations were determined using a bicinchoninic acid (BCA) protein assay kit (Beyotime, #P0010). Equal amounts of protein (20–30 μg) were mixed with 5× loading buffer and denatured by boiling at 100°C for 10 min. The protein samples were separated by SDS–polyacrylamide gel electrophoresis (SDS-PAGE) and subsequently transferred onto polyvinylidene fluoride (PVDF) membranes (Millipore). The membranes were blocked with 5% (w/v) skim milk in Tris-buffered saline containing 0.1% Tween-20 (TBST) at room temperature for 1 h and then incubated with primary antibodies overnight at 4°C. The primary antibodies used were as follows: KEAP1 (CST, #8047, 1:1000), NRF2 (CST, #12721, 1:1000), xCT (CST, #28001, 1:1000), FTH1 (CST, #3998, 1:1000), GPX4 (CST, #52455, 1:1000), STING (CST, #13647, 1:1000), phospho-STING (CST, #19781, 1:1000), IRF3 (CST, #4302, 1:1000), phospho-IRF3 (CST, #79945, 1:1000), TBK1 (CST, #3504, 1:1000), and phospho-TBK1 (CST, #5483, 1:1000). GAPDH (CST, #5174, 1:1000) or β-actin (CST, #4970, 1:1000) was used as the internal loading control. Following primary antibody incubation, the membranes were washed three times with TBST (10 min per wash) and incubated with horseradish peroxidase (HRP)-conjugated secondary antibodies (goat anti-rabbit IgG or goat anti-mouse IgG, 1:5000) at room temperature for 1 h. After extensive washing with TBST, protein bands were visualized using an enhanced chemiluminescence (ECL) detection kit (Millipore) and imaged with a Tanon 5200 imaging system. Band intensities were quantified using ImageJ software and normalized to the corresponding internal control.

**Real-Time Quantitative PCR (RT-qPCR) Analysis**

PAN02 cells in the logarithmic growth phase were seeded into 6-well plates at a density of 5 × 10^5^ cells per well and allowed to adhere overnight. The cells were then co-incubated with different hydrogel formulations, including FS-Gel, Zn-Gel, ZnFe-Gel, and G@ZnFe-Gel, for 6 h. After treatment, the culture medium was removed, and the cells were gently washed twice with ice-cold phosphate-buffered saline (PBS). Cells were subsequently detached using trypsin, and cell pellets were collected by centrifugation. Total RNA was extracted using a commercial RNA extraction kit (Yeasen, #18600ES) according to the manufacturer’s instructions. The concentration and purity of RNA were determined by ultraviolet spectrophotometry. For complementary DNA (cDNA) synthesis, 1 μg of total RNA from each sample was reverse-transcribed using Hifair® III First Strand cDNA Synthesis SuperMix (Yeasen) following the manufacturer’s recommended protocol. Real-time quantitative PCR was performed using Hieff UNICON® Universal Blue SYBR Green Master Mix (Yeasen) on a QuantStudio real-time PCR system. β-actin was used as the internal reference gene for normalization of target gene expression. The analyzed genes included ACSL4, IFIT1, IFIT3, IL-1β, IFN-β，LPCAT1, NRF2, p53, GPX4, IL-6, and TNF-α. The primer sequences for all target genes were provided in the **Table S1**. Relative mRNA expression levels were calculated using the 2^-ΔΔCt method. All reactions were performed in triplicate, and each experiment was independently repeated at least three times.

**MTT Rescue Assay with Fer-1 and RU.521**

PAN02 cells were treated with FS-Gel, Zn-Gel, ZnFe-Gel, or G@ZnFe-Gel (4 mg mL^-1^) in the absence or presence of ferrostatin-1 (Fer-1, 10 μM) or the cGAS inhibitor RU.521 (25 μM) for 24 h. The corresponding groups without inhibitor addition were designated as the No Inhibitor controls. Cell viability was then evaluated using the MTT assay as described above to assess the protective effects of pharmacological inhibition on treatment-induced cytotoxicity.

**Western Blot Analysis of Ferroptosis-Related Proteins and TBK1 Phosphorylation**

To assess the effect of ferroptosis inhibition on G@ZnFe-Gel-induced TBK1 activation, PAN02 cells were treated with Control, G@ZnFe-Gel, Fer-1, or G@ZnFe-Gel + Fer-1. Following treatment, cells were collected and lysed to extract total proteins. Equal amounts of protein were separated by sodium dodecyl sulfate-polyacrylamide gel electrophoresis (SDS-PAGE) and transferred onto polyvinylidene fluoride (PVDF) membranes. After blocking, the membranes were incubated with primary antibodies against ferritin heavy chain 1 (FTH1), nuclear factor erythroid 2-related factor 2 (NRF2), cystine/glutamate antiporter (xCT), phosphorylated TANK-binding kinase 1 (p-TBK1), and total TBK1, followed by incubation with the corresponding secondary antibodies. GAPDH was used as the internal loading control. Protein bands were visualized using an enhanced chemiluminescence (ECL) detection system, and their relative intensities were quantified by densitometric analysis. The expression levels of FTH1, NRF2, and xCT were normalized to GAPDH, whereas TBK1 phosphorylation was evaluated as the p-TBK1/TBK1 ratio.

**RT-qPCR Analysis of Type I Interferon-Related Gene Expression**

To further evaluate the contribution of ferroptosis-associated processes to downstream type I interferon signaling, PAN02 cells were divided into four groups and treated with PBS (Control), G@ZnFe-Gel, Fer-1, or G@ZnFe-Gel combined with Fer-1. Following treatment, total RNA was extracted from the collected cells and reverse-transcribed into complementary DNA (cDNA). Quantitative real-time polymerase chain reaction (RT-qPCR) was subsequently performed to determine the mRNA expression levels of interferon-β (IFN-β) and interferon-induced protein with tetratricopeptide repeats 3 (IFIT3). β-Actin was used as the internal reference gene, and relative mRNA expression levels were calculated using the 2^−ΔΔCt method. The expression levels were normalized to those of the PBS-treated control group to assess the effects of G@ZnFe-Gel and Fer-1 on downstream interferon-related transcriptional responses. All reactions were performed in triplicate, and experiments were independently repeated at least three times.

**Analysis of Peripheral Blood Immune Cells in Mice**

Seven-week-old male C57BL/6J mice (18–20 g, Jinan Pengyue Experimental Animal Breeding Co., Ltd.) were acclimatized for one week under standard laboratory conditions prior to experimentation. Mice were then randomly assigned to four groups (n = 5 per group): FS-Gel, Zn-Gel, ZnFe-Gel, and G@ZnFe-Gel. Each group received intraperitoneal injections of the corresponding formulation (100 μL per dose) once daily at a fixed time for five consecutive days. Twenty-four hours after the final administration, peripheral blood samples were collected into EDTA-K2–coated anticoagulant tubes. Whole blood was diluted with 1× red blood cell lysis buffer (Solarbio, #R1010) at a ratio of 1:10 and incubated at room temperature in the dark for 15 min to achieve complete erythrocyte lysis. Following lysis, leukocytes were harvested by centrifugation and resuspended in FACS buffer. For immunophenotyping, the cell suspensions were incubated with a panel of fluorochrome-conjugated antibodies at 4°C in the dark for 30 min. All antibodies were purchased from BD Biosciences and included CD45-APC-Cy7 (pan-leukocyte marker, #561037), CD19-FITC (B cell marker, #561740), CD3-PE (T cell marker, #561824), and CD11b-PerCP-Cy5.5 (macrophage marker, #550993). After staining, cells were washed twice with FACS buffer (300 × g, 5 min) to remove unbound antibodies and subsequently fixed in PBS containing 1% paraformaldehyde. Flow cytometric analysis was performed using a BD FACS Aria III flow cytometer. Data were analyzed by first gating on CD45^+^ leukocytes, followed by quantification of the relative proportions of B cells (CD19^+^), T cells (CD3^+^), and macrophages (CD11b^+^) within the CD45^+^ population.

**Transcriptome Sequencing Analysis**

PAN02 cells in the logarithmic growth phase were seeded in 6-well plates and treated with sterile PBS (negative control, Gibco, #10010023) or G@ZnFe-Gel for 6 h. Following treatment, the culture medium was removed, and cells were gently washed twice with PBS. Total RNA was extracted from each well using 1 mL of TRI-zol reagent (Invitrogen, #15596026) in accordance with the manufacturer’s protocol. RNA concentration and purity were determined using a Nano Drop 2000 spectrophotometer (Thermo Scientific), while RNA integrity was assessed using an Agilent 2100 Bioanalyzer with the RNA Nano 6000 Assay Kit (Agilent, #5067-1511). Only samples with an RNA integrity number (RIN) > 7.0 were used for subsequent analysis. Qualified RNA samples were submitted to Novogene (Tianjin, China) for library construction and high-throughput sequencing. Sequencing libraries were prepared using the NEBNext® Ultra^TM^ II Directional RNA Library Prep Kit (NEB), followed by paired-end sequencing (PE150) on an Illumina NovaSeq 6000 platform. Raw sequencing reads were subjected to quality control using FastQC and Trimmomatic to remove low-quality bases and adaptor sequences. The resulting clean reads were aligned to the mouse reference genome (GRCm39) using STAR aligner, and gene-level read counts were generated with feature Counts. Differential gene expression analysis was performed using the DESeq2 package (v1.36.0) in R. Genes with an absolute log₂ fold change (|log_2_FoldChange|) ≥ 1 and a false discovery rate (FDR) < 0.05 were considered significantly differentially expressed.

**Evaluation of *In Vivo* Antitumor Efficacy and Mechanistic Studies in a Pancreatic Cancer Mouse Model**

PAN02 murine pancreatic cancer cells in the logarithmic growth phase (5 × 10^6^ cells per mouse) were subcutaneously inoculated into the right flank of 6–8-week-old male C57BL/6 mice. When the tumor volume reached approximately 50 mm^3^, mice were randomly assigned to five treatment groups (n = 5 per group): Saline, FS-Gel, Zn-Gel, ZnFe-Gel, and G@ZnFe-Gel. Mice in each group received intratumoral injections of the corresponding formulation (100 μL per injection) every two days for a total treatment duration of 20 days. Throughout the treatment period, body weight was recorded every two days as an indicator of systemic toxicity. Tumor length (L) and width (W) were measured using a digital caliper, and tumor volume (V) was calculated according to the formula V = (L × W^2^)/2. Tumor growth curves were plotted accordingly. At the experimental endpoint, mice were euthanized, and tumors were excised, photographed, and weighed. Tumor tissues were fixed in 4% paraformaldehyde for 24 h, followed by routine gradient dehydration, paraffin embedding, and preparation of consecutive sections (4-5 μm thickness) for histological and immunological analyses.

Hematoxylin and eosin (H&E) staining was performed to evaluate tumor architecture, cellular morphology, and pathological alterations. Terminal deoxynucleotidyl transferase dUTP nick-end labeling (TUNEL) staining was conducted to assess DNA fragmentation and apoptotic cell death, while Ki-67 immunostaining was used to quantify tumor cell proliferation. Intratumoral reactive oxygen species (ROS) generation and spatial distribution were visualized using ROS-sensitive fluorescent probes (e.g., DCFH-DA). To elucidate ferroptosis induction and innate immune activation, immunofluorescence co-staining of GPX4 (a core ferroptosis regulator) and STING was performed to assess their expression levels and spatial correlation within tumor tissues. Immunogenic cell death (ICD) was evaluated by immunofluorescence co-staining of calreticulin (CRT, an early ICD marker) and high-mobility group box 1 (HMGB1, a late ICD marker). Adaptive immune responses were assessed by immunofluorescence co-staining of CD8 and CD4 to quantify the infiltration and distribution of cytotoxic T lymphocytes and helper T cells, respectively. Functional activation of intratumoral immune cells was further evaluated by immunofluorescence staining of interferon-γ (IFN-γ). In addition, immunohistochemical staining of interferon-β (IFN-β) was performed to assess the activation of type I interferon–mediated innate immune responses. Tumor-associated macrophage (TAM) polarization was analyzed by immunofluorescence co-staining of CD86 (M1-like macrophage marker) and CD163 (M2-like macrophage marker).

All animals were housed under standard laboratory conditions with free access to food and water. All animal procedures were conducted in strict accordance with the Guide for the Care and Use of Laboratory Animals and relevant National Research Council guidelines, and were approved by the Animal Ethics Committee of Qingdao University of Science and Technology (Approval No. QKDLL-2024-51).

***In Vivo* Biosafety Assessment**

To comprehensively evaluate the *in vivo* biosafety of the different treatment formulations, peripheral blood samples were collected from mice *via* retro-orbital bleeding on day 18 of treatment. Blood samples were centrifuged at 6000 rpm for 10 min at 4°C to obtain serum for biochemical analysis. Serum levels of alanine aminotransferase (ALT) and aspartate aminotransferase (AST) were measured to assess hepatic function, while blood urea nitrogen (BUN) and creatinine (CRE) levels were determined to evaluate renal function.

Following blood collection, mice were euthanized, and major organs, including the heart, liver, spleen, lungs, and kidneys, were carefully harvested. The collected organs were fixed in 4% paraformaldehyde, processed by routine paraffin embedding, and sectioned for histological examination. H&E staining was performed to assess tissue architecture and cellular morphology. Potential pathological alterations, including inflammatory cell infiltration, tissue necrosis, and structural disorganization, were systematically examined to evaluate treatment-related organ toxicity and overall *in vivo* biosafety.

**Statistical Analysis**

All quantitative data were presented as mean ± standard deviation (SD). Statistical analyses were performed using GraphPad Prism 8.5 software. Comparisons among multiple groups were conducted using one-way analysis of variance (ANOVA), while comparisons between two groups were performed using an unpaired Student’s *t*-test. A *P* value of less than 0.05 was considered statistically significant.

**Table S1 Gene prism**

| Gene Name | Forwad Primer（5’-3’）; Reverse Primer（5’-3’） |
| --- | --- |
| β‑actin | F:AGGTCGGTGTGAACGGATTTG；  R:GGGGTCGTTGATGGCAACA |
| ACSL4 | F:TCCTCCAAGTAGACCAACCCC；  R:AGTCCAGGGATACGTTCACAC |
| IFIT1 | F:GTCCGGTTAAATCCAGAAGATCC；R:GCTTTGTCTACGCGATGTTTCC |
| IFIT3 | F:TCAGGCTTACGTTGACAAGGT；  R:CACACTTTAGGCGTGTCCATC |
| IL-1b | F:AGCTACGAATCTCCGACCAC；  R:CGTTATCCCATGTGTCGAAGAA |
| LPCAT1 | F:GGCTCCTGTTCGCTGCTTT；  R:TTCCTCCATAAGGCCAGGGG |
| NRF2 | F:ACTACAGTCCCAGCAGAGTGA；  R:ACACTGGTCACACTGGGTTTT |
| p53 | F:GAGGTTGGCTCTGACTGTACC；  R:TCCGTCCCAGTAGATTACCAC |
| GPX4 | F:AGTACAGGGGTTTCGTGTGC；  R:CATGCAGATCGACTAGCTGAG |
| IL-6 | F：TCTATACCACTTCACAAGTCGGA；  R: GAATTGCCATTGCACAACTCTTT |
| IFN-β | F：CCCTATGGAGATGACGGAGA；  R: CTGTCTGCTGGTGGAGTTCA |
| TNF-α | F：CCTGTAGCCCACGTCGTAG；  R:GGGAGTAGACAAGGTACAACCC |

**Fig. S1**. SEM images of Zn-ZIF NPs, ZnFe-ZIF NPs, and G@ZnFe-ZIF NPs.

**Fig. S2**. Particle size distribution of Zn-ZIF NPs, ZnFe-ZIF NPs, and G@ZnFe-ZIF NPs.

**Fig. S3**. Time-dependent TMB chromogenic spectra of G@ZnFe NPs (reacted with Glu at room temperature). The inset photographs illustrate the corresponding color changes of the solution at each time point.

**Fig. S4.**  Enzymatic activity and long-term stability of immobilized GOD. (a) Time-dependent glucose consumption by free GOD and G@ZnFe-ZIF under identical reaction conditions at 2, 4, and 6 h. (b) Residual enzymatic activity of free GOD and G@ZnFe-ZIF after incubation in PBS containing 10% FBS at 37°C for 0, 12, 24, 48 h. The activity at 0 h for each formulation was independently normalized to 100%. Data are presented as mean ± SD (n = 3).

To evaluate the catalytic activity of GOD following immobilization. Free GOD and G@ZnFe-ZIF containing equivalent amounts of GOD were incubated in PBS (pH 7.4) containing 1 mg mL^-1^ glucose. As shown in **Fig. S4a**, both formulations exhibited progressive glucose consumption over 6 h, with comparable catalytic profiles. These results indicate that immobilization within ZnFe-ZIF preserved GOD-mediated glucose consumption without substantially compromising its catalytic performance under the tested conditions.

Next, to evaluate enzymatic stability under conditions that mimic aspects of the *in vivo* environment, free GOD and G@ZnFe-ZIF containing equivalent amounts of GOD were pre-incubated in PBS supplemented with 10% FBS at 37°C for 0, 12, 24, and 48 h. As shown in **Fig. S4b**, both formulations exhibited a gradual decline in residual activity during incubation; however, G@ZnFe-ZIF consistently retained higher activity than free GOD. After 48 h, G@ZnFe-ZIF retained approximately 82.6% of its initial activity, compared with 52.0% for free GOD. These results demonstrate that ZnFe-ZIF immobilization improves GOD activity retention over 48 h under the tested conditions. This enhanced stability may be attributable to the protective microenvironment provided by the MOF structures, consistent with previous reports on enzyme stabilization through nano-/MOF-based immobilization [1-3].

**Fig. S5**. Macroscopic appearance of FS-Gel, Zn-Gel, and ZnFe-Gel evaluated by the vial-inversion test at low (4°C) and elevated (>30°C) temperatures. All formulations exhibited a free-flowing sol state at 4°C and underwent rapid sol–gel transition to form self-supporting hydrogels within approximately 3 min upon heating above 30°C, demonstrating pronounced thermos-responsive gelation behavior.

**Fig. S6.** Schematic illustration of the Ca^2+^-responsive gelation mechanism of FS-Gel.

**Fig. S7.** Rheological frequency sweep analysis of G@ZnFe-Gel. The variation of storage modulus (G') and loss modulus (G'') with angular frequency (0.1-20 Hz).

**Fig. S8.** Quantitative analysis of fluorescence signals of G@ZnFe-Gel in subcutaneous tissues of mice.

**Fig. S9.** Validation of intracellular ROS detection in PAN02 cells. Representative bright-field, DCF fluorescence, and merged images of PAN02 cells following different treatments, including an H_2_O_2_-treated ROS-positive control.

**Fig. S10.** Cell cycle analysis of PAN02 tumor cells treated with various hydrogel formulations, including Control (untreated cells), FS-Gel, Zn-Gel, ZnFe-Gel, and G@ZnFe-Gel.

Flow cytometric analysis revealed that G@ZnFe-Gel markedly altered cell cycle progression in PAN02 cells (**Fig. S10**). While the Control and FS-Gel groups showed comparable cell cycle distributions, Zn-Gel and ZnFe-Gel induced only modest perturbations. In contrast, G@ZnFe-Gel triggered a pronounced G2/M phase arrest, characterized by a sharp increase in the G2/M population (51.5%) accompanied by a substantial reduction in G1-phase cells. This robust G2/M arrest indicates severe disruption of mitotic progression, providing an additional mechanistic basis for the potent antiproliferative and cytotoxic effects of G@ZnFe-Gel *in vitro* [4].

**Fig. S11.** Quantitative analysis of western blotting for key proteins related to ferroptosis. ****P* < 0.001, *****P* < 0.0001.

**Fig. S12.** Quantitative analysis of western blotting for proteins related to the cGAS-STING signaling. ****P* < 0.001, *****P* < 0.0001.

**Fig. S13.** Relative IFN-β mRNA expression in macrophages following different treatments. Data are presented as mean ± SD (n = 3). **P* < 0.05, ***P* < 0.01, ****P* < 0.001.

**Fig. S14.** Cell viability of PAN02 cells after treatment with different hydrogel formulations in the absence or presence of ferrostatin-1 (Fer-1, 10 μM) or RU.521 (25 μM). Data are presented as mean ± SD (n = 5). ****P* < 0.001, *****P* < 0.0001.

**Fig. S15.** (a) Western blot analysis of FTH1, NRF2, x-CT, p-TBK1, and TBK1 in PAN02 cells under different treatments (Control, G@ZnFe-Gel, Fer-1, and G@ZnFe-Gel + Fer-1). GAPDH was used as the loading control. (b-e) Quantitative analysis of FTH1 (b), NRF2 (c), x-CT (d), and p-TBK1 (e) protein expression.

**Fig. S16.**  Effects of ferrostatin-1 on G@ZnFe-Gel-induced interferon-related gene expression in PAN02 cells. RT-qPCR analysis of (a) IFIT3 and (b) IFN-β mRNA expression in the Control, G@ZnFe-Gel, Fer-1, and G@ZnFe-Gel + Fer-1 groups. Expression levels were normalized to β-actin. Data are presented as mean ± SD (n = 3). **P* < 0.05, ****P* < 0.001.

**Fig. S17.** qRT-PCR analysis of pro-inflammatory, interferon-stimulated, and ferroptosis-related gene expression in PAN02 cells following different treatments (Control, FS-Gel, Zn-Gel, and G@ZnFe-Gel). **P* < 0.05, ***P* < 0.01, ****P* < 0.001, *****P* < 0.0001.

The qRT-PCR analysis (**Fig. S13 and S14**) revealed that G@ZnFe-Gel treatment markedly upregulated genes associated with inflammation, innate immunity, and ferroptosis in PAN02 cells compared with all control formulations. Specifically, interferon-stimulated genes (IFN-β, IFIT1 and IFIT3) were most strongly induced in the G@ZnFe-Gel group, indicating robust activation of the cGAS-STING–mediated immune response [5]. In parallel, key ferroptosis-related genes, including LPCAT1 and ACSL4, were significantly elevated, together with oxidative stress–responsive regulators NRF2 and FTH1. These results demonstrated that G@ZnFe-Gel concurrently promoted ferroptotic vulnerability and innated immune activation at the transcriptional level.

**Fig. S18.** Quantitative fluorescence analysis of TUNEL staining. **P* < 0.05, ** *P* < 0.01, *** *P* < 0.001, **** *P* < 0.0001.

**Fig. S19.**  *In vivo* evaluation of lipid peroxidation induced by G@ZnFe-Gel. (a) Quantitative analysis of lipid peroxidation (LPO) levels in tumor tissue homogenates. (b) Measurement of malondialdehyde (MDA) content in tumor tissues. **P* < 0.05, ** *P* < 0.01, *** *P* < 0.001.

**Fig. S20.** Immunohistochemical staining of IFN-β in tumor tissues from different treatment groups.

**Fig. S21.** Immunofluorescence staining of tumor-associated macrophage (TAM) phenotypes in tumor tissue. Cell nuclei were stained with DAPI (blue). The M2-type TAM marker CD163 is shown in green fluorescence, and the M1-type TAM marker CD86 is shown in red fluorescence.

**Fig. S22.** Quantitative analysis of CD86 and CD163 protein expression levels. **P* < 0.05, ** *P* < 0.01, *** *P* < 0.001, **** *P* < 0.0001.

# References

[1] Fan R, Cai L, Liu H, et al. Enhancing metformin-induced tumor metabolism destruction by glucose oxidase for triple-combination therapy. ***J. Pharm. Anal.*** 2024, 14, 321-334.

[2] Zhang W, Lin W, Li Z, et al. Glucose oxidase and conjugated polymer nanocomplexes for synergistic photothermal/starvation/oxidation therapy. ***Mater. Chem. Front.*** 2023, 7, 3706-3713.

[3] Wu X, Ge J, Yang C, et al. Facile synthesis of multiple enzyme-containing metal–organic frameworks in a biomolecule-friendly environment. ***Chem. Commun.*** 2015, 51, 13408–13411.

[4] Guan T, Chen Z, Wang X, et al. Harnessing Mn^2+^ ions and antitumor peptides: a robust hydrogel for enhanced tumor immunotherapy. ***J. Am. Chem. Soc.*** 2025, 147, 6523-6535.

[5] Nan Y, Cui D, Guo J, et al. STING COPII ER export trafficking and signaling primed by phosphorylation switches. ***Adv. Sci.*** 2025, 12, e03660.
